# Dissecting the pathways coordinating patterning and growth by plant boundary domains

**DOI:** 10.1371/journal.pgen.1007913

**Published:** 2019-01-24

**Authors:** Aude Maugarny-Calès, Millán Cortizo, Bernard Adroher, Nero Borrega, Beatriz Gonçalves, Geraldine Brunoud, Teva Vernoux, Nicolas Arnaud, Patrick Laufs

**Affiliations:** 1 Institut Jean-Pierre Bourgin, INRA, AgroParisTech, CNRS, Université Paris-Saclay, France; 2 Univ. Paris-Sud, Universite Paris-Saclay, Orsay, France; 3 Laboratoire de Reproduction et de Développement des Plantes, INRA, CNRS, ENS de Lyon, UCB Lyon 1, Université de Lyon, France; "USDA-ARS Pacific West Area", UNITED STATES

## Abstract

Boundary domains play important roles during morphogenesis in plants and animals, but how they contribute to patterning and growth coordination in plants is not understood. The *CUC* genes determine the boundary domains in the aerial part of the plants and, in particular, they have a conserved role in regulating leaf complexity across Angiosperms. Here, we used tooth formation at the Arabidopsis leaf margin controlled by the CUC2 transcription factor to untangle intertwined events during boundary-controlled morphogenesis in plants. Combining conditional restoration of CUC2 function with morphometrics as well as quantification of gene expression and hormone signaling, we first established that tooth morphogenesis involves a patterning phase and a growth phase. These phases can be separated, as patterning requires CUC2 while growth can occur independently of CUC2. Next, we show that CUC2 acts as a trigger to promote growth through the activation of three functional relays. In particular, we show that *KLUH* acts downstream of CUC2 to modulate auxin response and that expressing KLUH can compensate for deficient CUC2 expression during tooth growth. Together, we reveal a genetic and molecular network that allows coordination of patterning and growth by CUC2-defined boundaries during morphogenesis at the leaf margin.

## Author summary

During organogenesis, patterning, the definition of functional subdomains, has to be strictly coordinated with growth. How this is achieved is still an open question. In plants, boundary domains are established between neighboring outgrowing structures and play a role not only in the separation of these structures but also in their formation. To further understand how these boundary domains control morphogenesis, we used as a model system the formation of small teeth along the leaf margin of Arabidopsis, which is controlled by the *CUP-SHAPED COTYLEDON2* (*CUC2*) boundary gene. The *CUC* genes determine the boundary domains in the aerial part of the plants and in particular they have been shown to have a conserved role in regulating serration and leaflet formation across Angiosperms and thus are at the root of patterning in diverse leaf types. We manipulated the expression of this gene using an inducible gene expression that allowed restoration of *CUC2* expression in its own domain at different developmental stages and for different durations, and followed the effects on patterning and growth. Thus, we showed that while *CUC2* is required for patterning it is dispensable for sustained growth of the teeth, acting as a trigger for growth by the activation of several functional relays. We further showed that these findings are not specific to the inducible restoration of *CUC2* function by analyzing multiple mutants.

## Introduction

In all multicellular organisms, morphogenesis relies on the tight control of two intimately linked processes: patterning, which subdivides the tissues in groups of cells with different fates, and growth, which increases tissue size [1]. Such a coordination can be achieved in animals through the production of diffusible signals, or morphogens, that can both regulate proliferation and determine different cell fates in a concentration-dependent manner [2–4]. These morphogens are produced by specific groups of cells, called organizers, that are often located at the boundary between domains with different identities [5,6]. Morphogenesis in plants differs from animal development by several aspects as for instance no cell migration and only little cell death occur. Furthermore, the existence of morphogens in plants is still questioned although the plant hormone auxin, small RNAs or small peptides have been proposed to have a morphogen-like activity [7–9]. On the contrary, it has been clearly established that proper plant morphogenesis relies on the formation of functional boundary domains [10–12].

In plants, several types of boundaries have been described. For instance, in the developing leaf, a boundary lies at the junction of the adaxial and abaxial domains [13–16]. During early phases of leaf development, these two domains are directly adjacent and it is only later that interactions between these two domains lead to the formation of a third middle domain a few cells wide [17–19]. Another type of boundary domain is widely found in the aerial part of the plant and is directly related to the plant-specific mode of organogenesis that occurs at the shoot apical meristem. These inter-organ boundaries are narrow cellular domains located between an organ and either, the meristem from which it was initiated, or a neighboring organ. These domains are genetically defined by the expression of several genes. The central role of these boundary domains for plant development is demonstrated by the large range of developmental abnormalities observed following the inactivation of one or several of these genes [10–12]. Hence, mutants affected in boundary function show defects in the initiation of new growth axes: meristem formation is perturbed during the embryonic and post-embryonic phase, leading respectively to lack of a shoot apical meristem and branching defects [20–23] and placenta and ovule formation alterations [24–26]. In addition, boundary mutants exhibit fusions between organs such as adjacent floral organs or between inflorescence stem and floral pedicels [23,27], have a modified phyllotaxy during stem growth [27,28] and present reduced leaf shape complexity [29–32]. Because boundary domains are located in grooves between outgrowing structures, the cells they are formed of share specific characteristics. For instance, boundary cells show negative Gaussian curvature and experience a highly anisotropic mechanical stress that leads to the alignment of the cortical microtubule network along the main axis of the boundary domain [33–35]. In addition, growth of boundary cells is reduced and occurs preferentially parallel to the cortical microtubule network orientation while it is perpendicular in most plant cells [33,35,36]. This particular boundary growth pattern is associated with a depletion of several hormones from the boundary domain [11]. Indeed, auxin is depleted from boundary cells as a result from divergent distribution of the auxin efflux carrier PIN FORMED 1 (PIN1) [37,38] and boundary cells show reduced brassinosteroid signaling [39]. In turn, reduced auxin and brassinosteroid signaling, combined with mechanical stresses contribute to shape the specific expression pattern of boundary genes [39–43].

How boundaries control plant development has been initially analyzed in the context of meristem development and more recently of leaf shaping. Leaves are initiated as small primordia at the meristem periphery and go through a process of morphogenesis and growth to acquire their mature shape and size [44–46]. In particular, new growth axes can be formed at the leaf margin and will, depending on the species, develop into small outgrowths such as the teeth of the serrated Arabidopsis leaf, or larger structures such as the leaflets of the compound tomato leaf. Definition of a boundary domain at the leaf margin by the activity of transcription factors from the NO APICAL MERISTEM/CUP-SHAPED COTYLEDON 3 (NAM/CUC3) family is required for these marginal outgrowths to properly form [20,21,47]. For instance, in Arabidopsis, serration formation is affected in *cuc2* mutants while conversely more pronounced serrations are observed when *CUC2* expression levels are increased as a result of reduced activity of its regulatory miRNA, miR164 [29,48,49]. *CUC3* also contributes to serration development, while *CUC1*, the third member in Arabidopsis is not expressed and appears to have no role during leaf shaping [48]. More generally, reducing *NAM*/*CUC3* activity during leaf development leads not only to defects in marginal outgrowth separation, such as leaflet fusion, but also to patterning defects reflected by the abnormal number and position of leaflets [30–32,50]. Similar defects in the patterning of the leaf marginal outgrowth and separation are observed in mutants in which the formation of localized auxin response is perturbed [51–54]. Indeed, it has been shown that leaf marginal outgrowth formation relies on an interdependency between boundaries and auxin signaling: *CUC2* activity is required for building up discrete maxima of auxin response via a modification of polar auxin transport and, conversely, localized auxin response is required for proper *CUC2* expression [55,56]. In addition to auxin, other plant hormones, including gibberellic acid and cytokinins, contribute to leaf margin morphogenesis [57–59]. The *KLUH* gene, which encodes for a cytochrome P450 protein, extends cell proliferation duration, possibly through an unknown mobile signal distinct from the classical plant hormones [60–62]. Therefore, while leaf margin patterning appears to rely on the interplay between *CUC* genes and auxin signaling, which factors control later tooth outgrowth and how these patterning and growth processes are intertwined is not understood.

Here, we used tooth formation at the leaf margin as a model to dissect the mechanisms by which the inter-organ boundary domain coordinates patterning and growth to direct morphogenesis. To help separating linked events, we used conditional restoration of CUC2, a regulator of leaf boundary, to induce tooth formation and analyze the downstream molecular effects leading to morphogenesis. Using this system, we showed that a transient pulse of CUC2 expression is sufficient to trigger both patterning and growth of margin serrations, and characterized the contribution of three genetic or molecular actors that can act as functional relays for CUC2. The role of these actors revealed by the CUC2 conditional expression system was confirmed in wild-type tooth morphogenesis. In particular, we highlight the role of *KLUH* in relaying CUC2 as a promoter of tooth outgrowth and present evidence that it may act by modulating auxin response. Thus, we propose a sequential scenario accounting for the coordination of patterning and growth via a network activated by the CUC2 boundary gene during leaf serration.

## Results

### The *cuc2-1* and *cuc2-3* mutants differentially affect leaf margin development but do not allow separating CUC2 roles on patterning and growth

**Fig 1:**
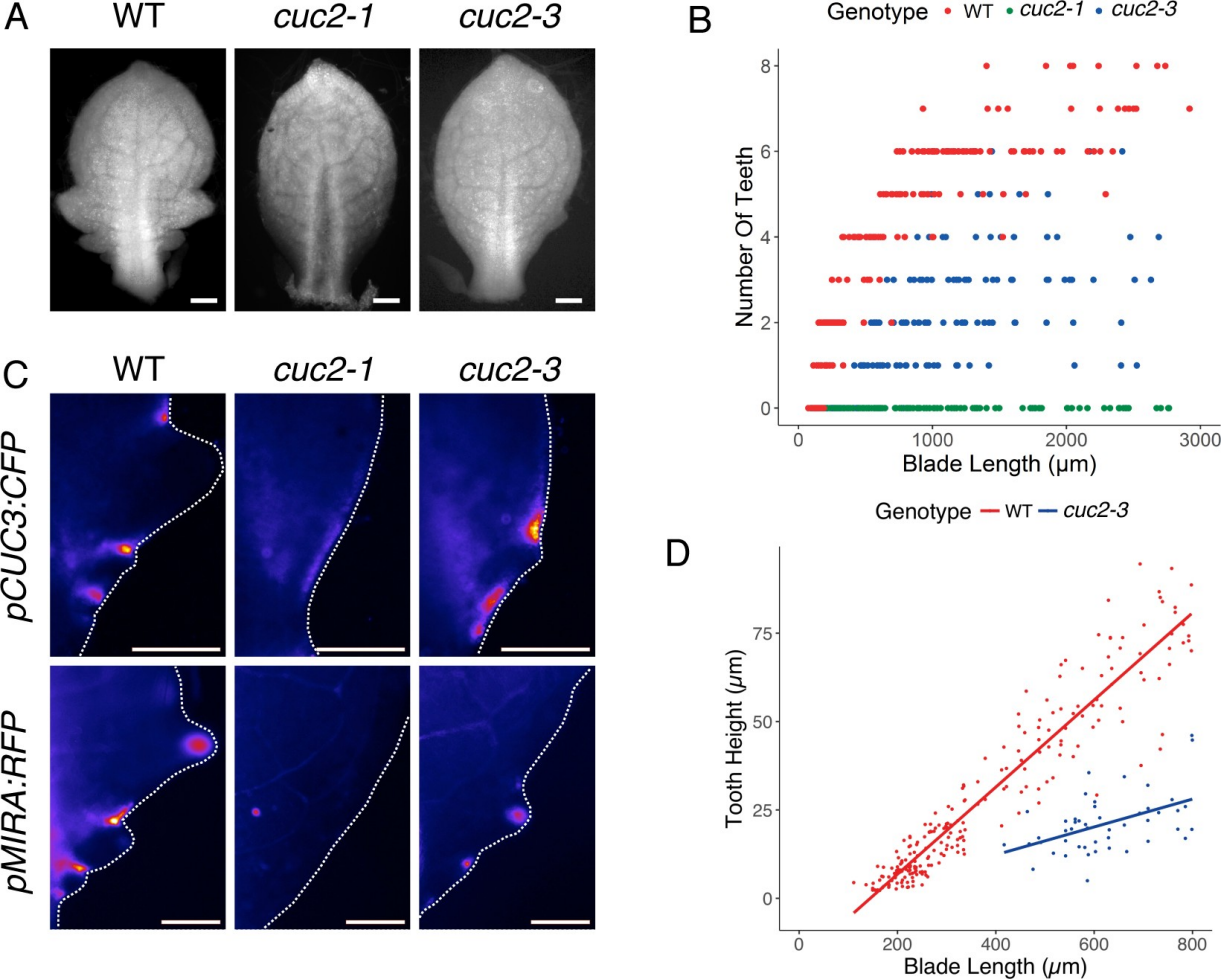
Patterning of the leaf margin is abolished in *cuc2-1* while *cuc2-3* shows leaf margin patterning and residual tooth growth. (A) Leaf primordia about 1mm-long of WT (Wild Type, Col-0) *cuc2-1* and *cuc2-3*. Note the irregular serration pattern in *cuc2-3*. (B) Number of teeth in WT, *cuc2-1* and *cuc2-3* primordia during development. Developmental stage is evaluated by measure of the blade length. (C) Expression pattern of *pCUC3*:*CFP* and *pMIR164A*:*RFP* in WT, *cuc2-1* and *cuc2-3*. (D) Tooth height evolution along blade length in WT and *cuc2-3*. The slope for WT is 0.123 and 0.039 for *cuc2-3*. Data in A and B are from [65] for WT and *cuc2-1*. Scale bars: 100 μm.

With the aim of trying to separate the contribution of *CUC2* during leaf margin patterning and tooth outgrowth, we reexamined the early leaf phenotype of two *cuc2* mutant alleles, *cuc2-1* and *cuc2-3* [29,48]. Both mutants have been described as developing leaves with smooth margins when grown under long day conditions [29,48]. Because leaf serration is more pronounced in plants grown in short-days, we reexamined the early leaf phenotype of *cuc2-1* and *cuc2-3* plants grown under these conditions. While leaf margins of *cuc2-1* were smooth, small teeth formed along *cuc2-3* primordia (Fig 1A and 1B). This phenotype difference prompted us to reexamine the molecular basis of the two mutations. *cuc2-1* carries a Tag1 transposon insertion in the first exon [21] while *cuc2-3* has a T-DNA insertion 99bp upstream of the ATG [23]. While CUC2 mRNA level was less than 5% of the wild type in *cuc2-1* it represented about 20% in *cuc2-3* (S1 Fig). Therefore *cuc2-3* is a hypomorphic allele of *CUC2* while *cuc2-1* is likely to be a true null allele. To more precisely characterize leaf margin patterning in these mutants, we introduced a *pCUC3*:*CFP* and a *pMIR164A*:*RFP* reporter in both genetic backgrounds. In the wild type, both reporters mark the boundary domain, while *pMIR164A*:*RFP* also marks the tip of the outgrowing teeth (Fig 1C) [29,48]. *pCUC3*:*CFP* showed a weak, continuous expression in *cuc2-1* while *pMIR164A*:*RFP* expression was not detected at the margin, suggesting that no patterning of the leaf margin associated with tooth formation occurred in the *cuc2-1* mutant (Fig 1C). Conversely, in *cuc2-3* the expression of both markers was discontinuous like in the wild type, albeit weaker, confirming that patterning of the leaf margin occurred (Fig 1C). Next, we quantified the rate of tooth outgrowth, which we found to be reduced to about 1/3 of the wild-type level in *cuc2-3* (Fig 1D). Therefore, this indicates that in *cuc2-1* both patterning and tooth outgrowth are compromised while in *cuc2-3* both processes occur, although growth is severely reduced. Altogether, this indicates that these mutants do not allow separating the roles of *CUC2* in patterning and growth.

### Manipulation of CUC2 expression timing shows that CUC2 is dispensable for tooth outgrowth

**Fig 2:**
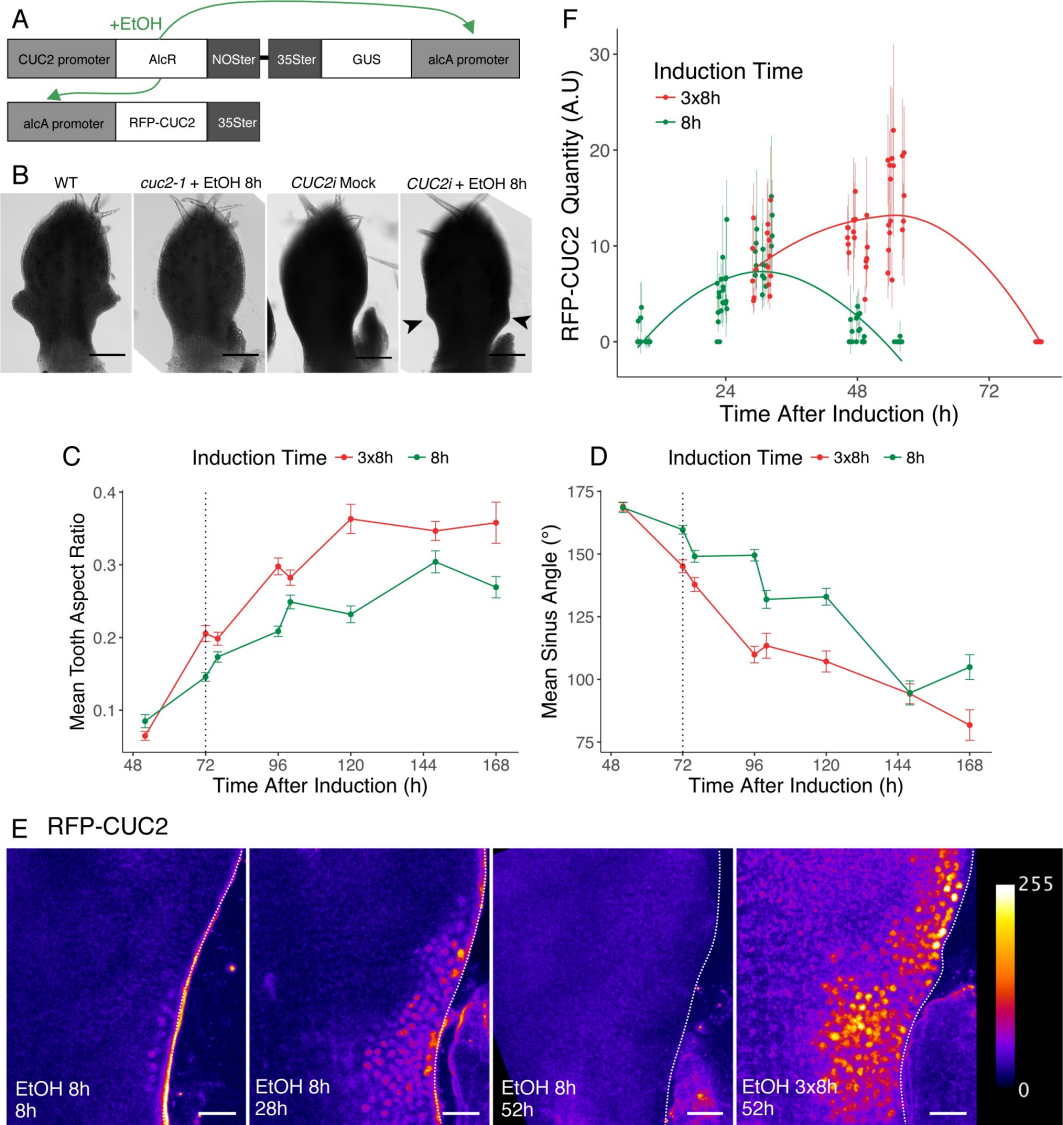
A pulse of CUC2 expression is sufficient to induce tooth formation. (A) Schematic representation of the construct present in the *CUC2i* line, allowing ethanol inducible expression of RFP-CUC2 in a *cuc2-1* mutant background. The AlcR protein is expressed under the control of the *CUC2* promoter and specifically binds to the *AlcA* promoters upon ethanol application. Thus, activated AlcR drives both *GUS* and *RFP-CUC2* transcription. (B) Young leaves of wild type (WT), *cuc2-1* treated with ethanol and *CUC2i* upon mock and ethanol treatment. Induced teeth are indicated by black arrowheads. *CUC2i* leaves are observed 4 days after treatment and a representative wild-type primordium of similar blade length is shown. (C-D) Tooth aspect ratio (C) and sinus angle (D) dynamics after 8h or 3x8h inductions. Data are mean ± SEM, tooth number *n* ≥ 11. The black dashed line indicates 72h, a time at which RFP-CUC2 is not detectable anymore following an 8h induction. (E) Dynamics of RFP-CUC2 pattern after 8h or 3x8h inductions in a *CUC2i* background. The time at which the sample was imaged following the start of induction is indicated. (F) Quantification of RFP-CUC2 fluorescence after single 8h and triple 8h inductions (designated 8h and 3x8h) of the *CUC2i* line. Each point is the mean ± SD of *n* = 10 nuclei per sinus. Scale bars: (B) 100μm and (E) 20μm.

Next, we tested whether the roles of CUC2 in patterning and growth could be separated by manipulating the timing of CUC2 expression. For this, we manipulated CUC2 expression timing using the ethanol-switch strategy [63,64] to produce inducible restoration of CUC2 expression under its own promoter for different durations. The ethanol-inducible construct containing an RFP-tagged version of CUC2 was introduced in *cuc2-1* and this line was designated *CUC2i* (Fig 2A). Ethanol induction restored tooth formation (Fig 2B and S2A Fig), but not all leaves could form teeth, as leaves that were longer than about 1200μm did not respond (S2B Fig). Increasing induction time lead to more teeth initiated at the margin (S2C Fig), suggesting that in *CUC2i*, multiple teeth are formed sequentially as in the wild type. Notably, an 8h induction was sufficient to form on average about one pair of teeth per leaf (Fig 2B and S2C Fig). To characterize CUC2-induced morphological changes at the leaf margin, we analyzed in parallel the evolution of two shape descriptors *Tooth Aspect Ratio* (reflecting anisotropic deformation during tooth growth) and *Sinus Angle* (reflecting local deformation due to growth repression at the sinus, see S2D Fig). Morphometric analyses revealed that, following an 8h induction, teeth started to emerge at 48h, with tooth aspect ratio and sinus angle continuously increasing and decreasing respectively until 168h (Fig 2C and 2D).

Next, we wanted to characterize how an 8h-long ethanol induction translates into CUC2 protein accumulation dynamics. For this, we took advantage of the RFP tag to characterize RFP-CUC2 pattern (Fig 2E and 2F) after an 8h induction. Eight hours after an 8h induction, RFP-CUC2 formed a continuous domain along the margin. At 28 hours after induction, RFP-CUC2 pattern started to become discontinuous (seen in 10/22 samples), while at 52h RFP-CUC2 could not be detected anymore. To be able to visualize RFP-CUC2 dynamics for a longer time, we next induced the *CUC2i* line 8h per day for 3 days (3x8h). Following such a 3x8h induction, RFP-CUC2 clearly resolved into discontinuous domains, with a higher expression at the sinuses of the small outgrowing tooth visible at 52h (Fig 2E), like observed for the expression of the translational reporter pCUC2:CUC2:VENUS (right panel in Fig 2E). Therefore, RFP-CUC2 expression resolved in about 48 hours after induction from a single domain to discontinuous domains, a feature required for CUC2 function during wild-type tooth morphogenesis [56]. Both quantifications of RFP-CUC2 fluorescence and real time RT-PCR showed that following an 8h induction RFP-CUC2 level transiently increased for about 24h-30h hours before decreasing and becoming undetectable between 48h to 72h (Fig 2F and S2E Fig). Repeated induction (3x8h) leads to higher and prolonged RFP-CUC2 levels that however became undetectable at 80h (Fig 2F).

Altogether, this indicates that following an 8h ethanol induction, CUC2 is transiently expressed and recapitulates the spatial dynamics observed during wild-type development (Fig 2E and 2F). This pulse of CUC2 expression is not only sufficient to trigger patterning of the leaf margin resulting in tooth initiation, but also for sustained tooth outgrowth that persists after CUC2 expression becomes undetectable (Fig 2C and 2D). We therefore conclude that CUC2 is sufficient to promote patterning at the leaf margin and is dispensable for later tooth growth, indicating that it acts as a trigger for tooth morphogenesis.

### CUC2 level drives tooth growth rate at the leaf margin

**Fig 3:**
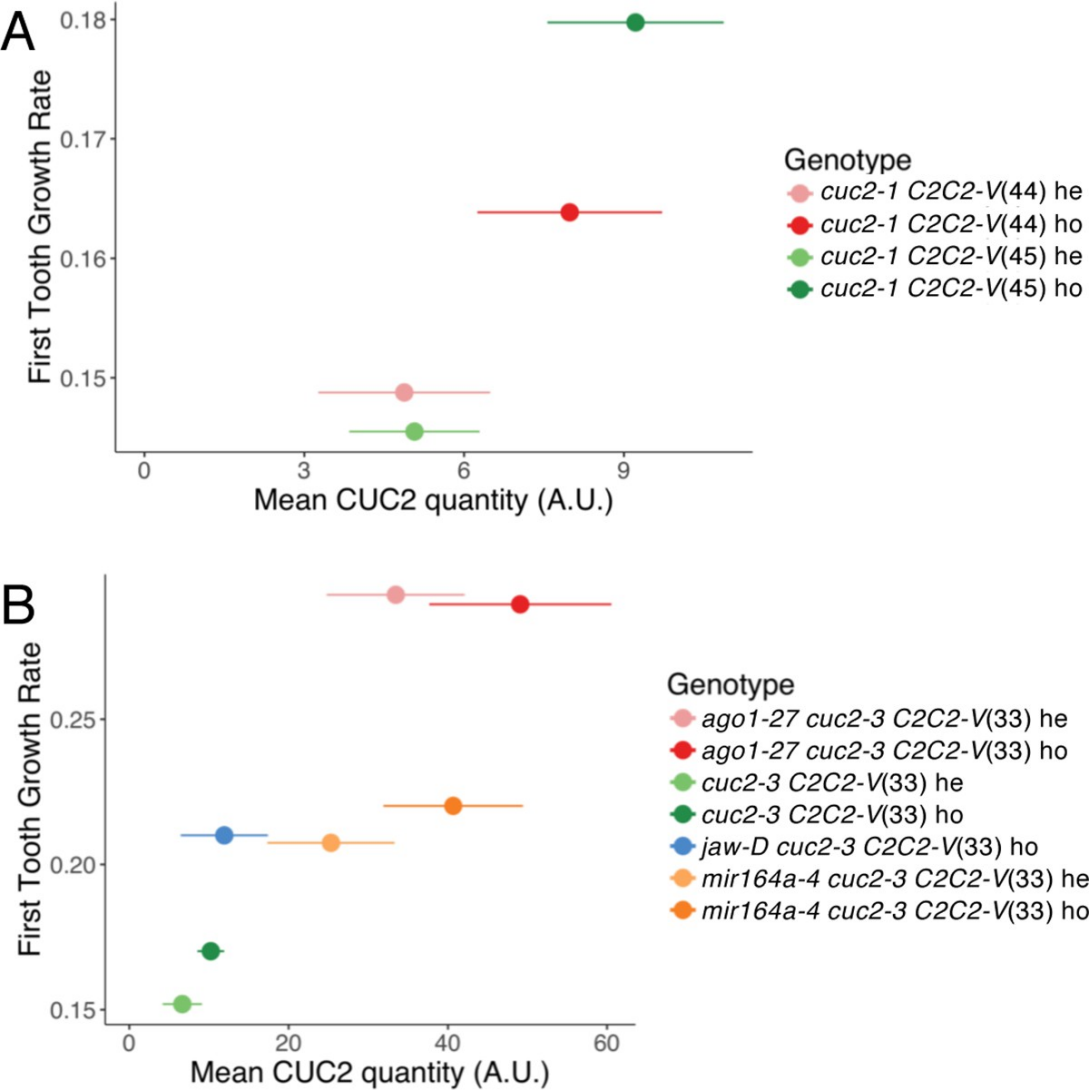
CUC2 levels determine tooth growth rate. Correlation between tooth growth rate and CUC2 levels. CUC2 levels and first tooth growth rate were measured in *cuc2-1* (A) or *cuc2-3* backgrounds (B). These lines were either homozygous (ho) or heterozygous (he) for a *pCUC2*:*CUC2*:*VENUS* construct (noted *C2C2-V*), inserted in one of three possible loci (named 33, 44 and 45) and could be combined with different genetic backgrounds (*mir164a-4* or *ago1-27*). CUC2 quantity is calculated for blade length between 400 and 600μm, (sinus number *n* ≥ 8), and is represented as mean ± SD (see S2 Fig for details). The Spearman’s rank correlation coefficients *r*_*s*_ are 0.80 and 0.85 for (A) and (B) respectively and the associated *p*-values are 0.02 and 0.33 for (A) and (B).

CUC2 may act as a trigger for tooth morphogenesis via two possible scenarii. It may act as a licensing factor allowing growth to occur. In this scenario, the growth rate would not be related to the level of CUC2 expression. Alternatively, CUC2 may promote growth which rate would therefore be linked to CUC2 level. Earlier observations indicating that the level of leaf serration in mature leaves is related to *CUC2* expression levels [29,48,49,55,56,65] do not allow to discriminate between these scenario as larger serrations in mature organs may result for instance from faster tooth growth or from prolonged growth. Therefore, to further characterize how CUC2 promotes growth we investigated the link between CUC2 protein levels and tooth growth rate. For this, we determined tooth growth rate by morphometrics and quantified the *CUC2-VENUS* signal in several *cuc2-1* or *cuc2-3* mutants complemented by a translational *pCUC2*:*CUC2-VENUS* reporter. Hence, a positive correlation between mean CUC2 protein levels and tooth growth rates was observed in four *cuc2-1* backgrounds that had different CUC2 expression levels as a result of different *pCUC2*:*CUC2-VENUS* integration site or copy number (Fig 3A and S3A and S3B Fig). This correlation was also observed in six *cuc2-3* backgrounds with a much wider range of *pCUC2*:*CUC2-VENUS* expression levels as a result of reduced miRNA inhibition of CUC2 level (Fig 3B and S3C and S3D Fig). Altogether, this indicated that CUC2 acts as a trigger that promotes tooth outgrowth in quantitative manner. A corollary of this observation is that CUC2 may activate one or several downstream factors that act as functional relays to maintain tooth outgrowth after CUC2 disappearance.

### *CUC3* acts as a local functional relay for CUC2-triggered tooth outgrowth

**Fig 4:**
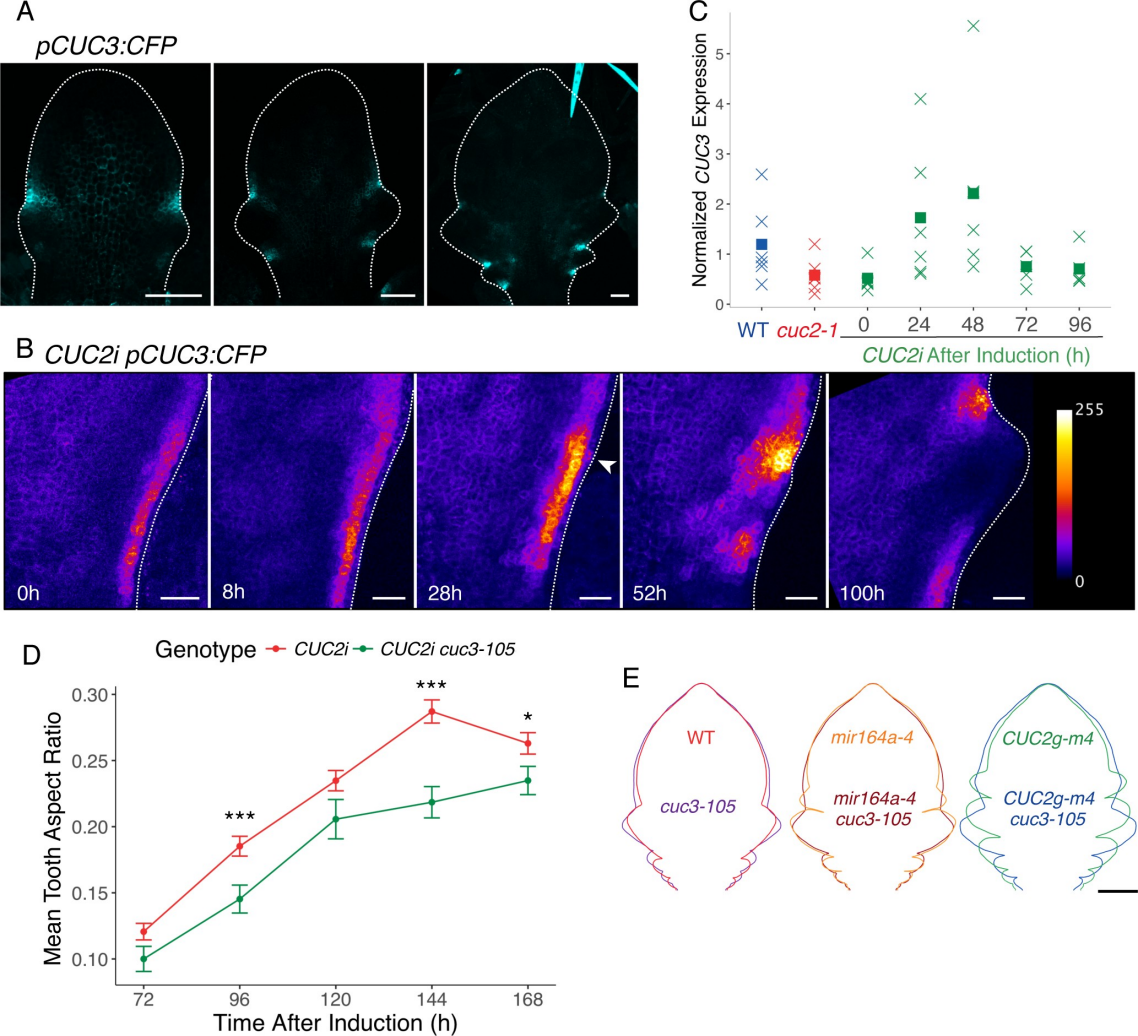
*CUC3* is a local functional relay for CUC2-triggered tooth outgrowth. (A) Expression of a *pCUC3*:*CFP* reporter during leaf development in a wild-type background. (B) Dynamics of *pCUC3*:*CFP* pattern in a *CUC2i* background after an 8h induction. Time following the start of induction is indicated. The white arrowhead points to epidermal *pCUC3*:*CFP* signal. Note that the 0h time-point corresponds to an un-induced control. (C) Real-time RT-PCR quantifications of *CUC3* in the wild type (WT), *cuc2-1* and *CUC2i* at 0 to 96 hours after an 8h ethanol induction. RNAs were extracted from microdissected leaf margins and *CUC3* levels are normalized by *EF1α* and *qREF*. Crosses represent individual data points while squares are mean of the different samples, sample number *n* ≥ 5. (D) Tooth aspect ratio dynamics after and 8h ethanol induction in a *CUC2i* and *CUC2i cuc3-105* background. Data are mean ± SEM (tooth number *n* ≥ 10). Statistical significance (Student’s test) is designated by * p<0.05, *** p<0.005. (E) Mean contours of 1100μm-long leaf 11, 12 and 13 WT/*cuc3-105*; *CUC2g-m4*/*CUC2g-m4 cuc3-105*; *mir164a-4*/*mir164a-4 cuc3-105*. Scale bars: (A) 50μm, (B) 20μm and (E) 200μm.

Because *CUC3* is partially redundant with *CUC2* in shoot development [23,26,27,47] and contributes to sustained serration outgrowth [48], *CUC3* appeared as one of the possible relay for CUC2 activity. This hypothesis is supported by the expression of the *pCUC3*:*CFP* transcriptional reporter in sinuses between emergent teeth in a pattern similar to *CUC2* (Fig 4A).

We first followed *pCUC3*:*CFP* expression in *CUC2i* after an 8h induction (Fig 4B). Before induction, *pCUC3*:*CFP* expression was low and continuous along the margin in the sub-epidermal layers, a pattern identical to the one observed in the *cuc2-1* background (Fig 1C). Next, at 28h after induction, *pCUC3*:*CFP* expression became more intense and was observed in some epidermal cells (observed in 12/22 samples) that had high RFP-CUC2 levels (S4A Fig). At 52h, *pCUC3*:*CFP* pattern became discontinuous, disappearing from the outgrowing tooth and finally being mostly restricted to its distal sinus at 100h (Fig 4B). The increase in *pCUC3*:*CFP* expression was confirmed by real time RT-PCR analysis on microdissected *CUC2i* leaf margin tissues that showed higher *CUC3* transcript levels at 24 and 48h after induction (Fig 4C).

Having shown that *CUC3* expression is modified by CUC2 induction, we next tested whether *CUC3* also acts as a functional relay for CUC2. For this, we compared morphometric parameters after ethanol induction in *CUC2i* and *CUC2i cuc3-105* backgrounds (Fig 4D and S4B Fig). The increase in tooth aspect ratio is delayed in *CUC2i cuc3-105* compared to the *CUC2i* control from 96h onwards and, although the sinus angle is initially identical, *CUC2i cuc3-105* show more open angles from 120h onwards (S4B Fig). Together, these analyses showed that *CUC3* contributes in a quantitative manner to tooth outgrowth following CUC2 induction.

**Fig 5:**
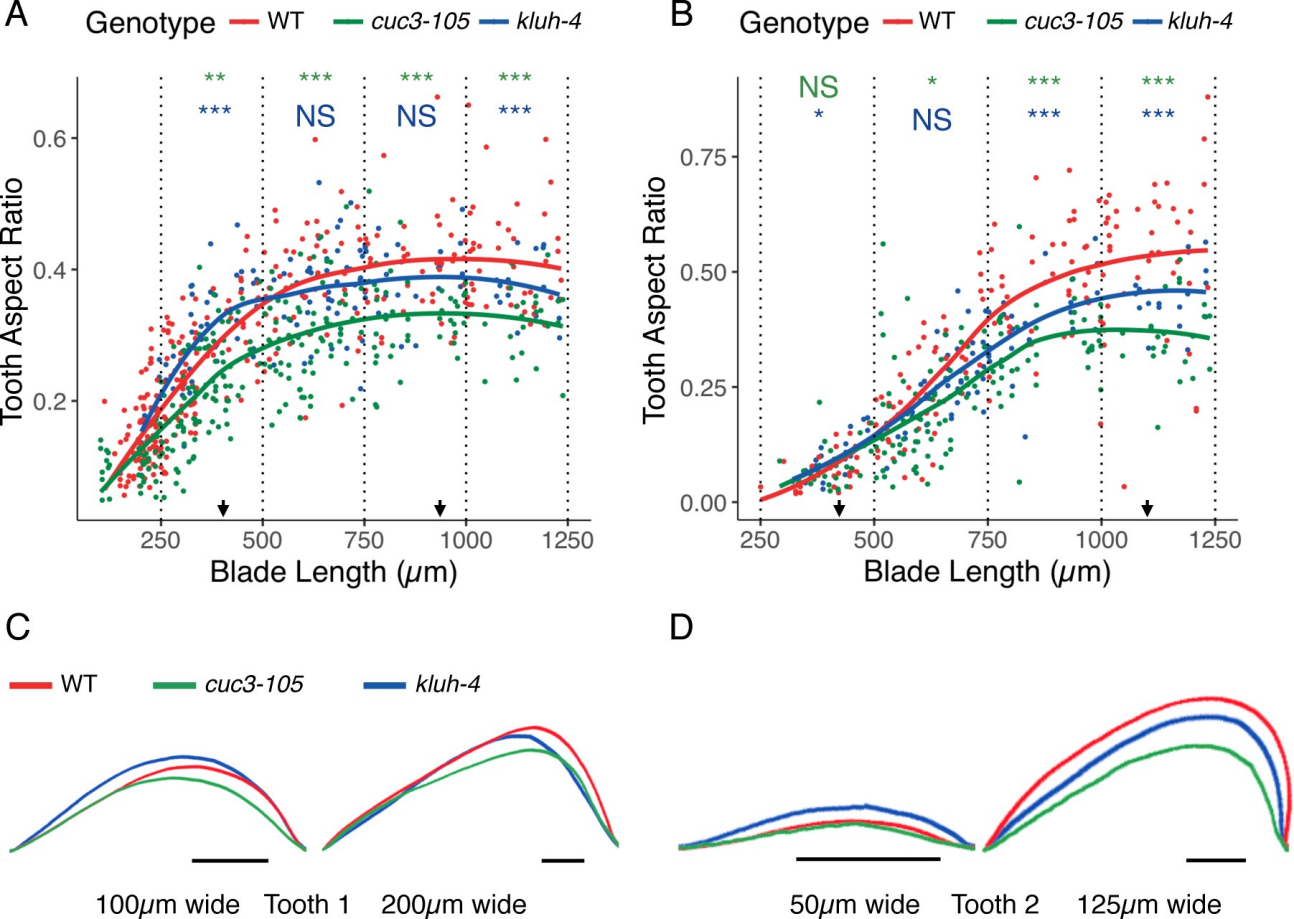
*CUC3* and *KLUH* are regulators of leaf morphogenesis. (A, B) Dynamics of tooth 1 (A) and tooth 2 (B) shape in wild type (WT), *cuc3-105* and *kluh-4* during early leaf morphogenesis. Data presented are measures of individual sinuses and a local regression is shown for each genotype, blade length is used as a proxy for the leaf developmental stage. Statistical significance for data grouped in 250μm-wide bins starting at 250μm is determined by Student’s test and is shown in color (green for *cuc3-105* compared to WT, blue for *kluh-4* compared to WT) for each bin (NS: not significant, * p< 0.05, ** p< 0.01, ***p< 0.005). (C, D) Mean tooth shape for tooth 1 (C) and tooth 2 (D) at two developmental stages of wild type (WT), *cuc3-105* and *kluh-4*. Arrows in A and B indicate the leaf blade sizes for which the mean tooth shapes are shown. Scale bars: 25μm.

Several observations suggested that the role of *CUC3* as a functional relay of CUC2 is not limited to the *CUC2i* line. First, parallel quantification of *CUC3* and *CUC2* promoter activities in tooth sinuses during wild-type leaf development revealed a strong correlation between them (S4C Fig). Second, three lines with increased CUC2 levels compared to wild type (*mir164a-4* and *ago1-27*, Fig 3B and *CUC2g-m4*) also presented an increase in *pCUC3*:*CFP* activity (S4D Fig). Third, precise morphometric analysis showed that in addition to a late defect in tooth growth already reported [48], *cuc3-105* tooth growth was also reduced early on during development compared to wild type (Fig 5A–5D, green compared to red). Indeed, tooth aspect ratio was significantly reduced in *cuc3-105* leaves with blades longer than 250μm for tooth 1 and longer than 500μm for tooth 2 (Fig 5, green compared to red). Fourth, the *cuc3-105* mutation partially suppressed the increased serration of *mir164a-4* and *CUC2g-m4* (Fig 4E and S4E Fig).

In conclusion, our results show that *CUC3* expression level and spatial dynamics are determined by CUC2 and that *CUC3* is required for tooth growth. Based on these observations and because *CUC3* is essentially expressed in the same domain as CUC2, we conclude that *CUC3* acts as a local functional relay for CUC2 activity during tooth growth.

### Localized high auxin response acts as a distant and long-lasting relay for CUC2-triggered tooth outgrowth

Because *CUC* expression interacts with auxin response during the formation of new growth axes [24,38,56,66] we investigated whether auxin response mediates CUC2 promoting effect on growth.

**Fig 6:**
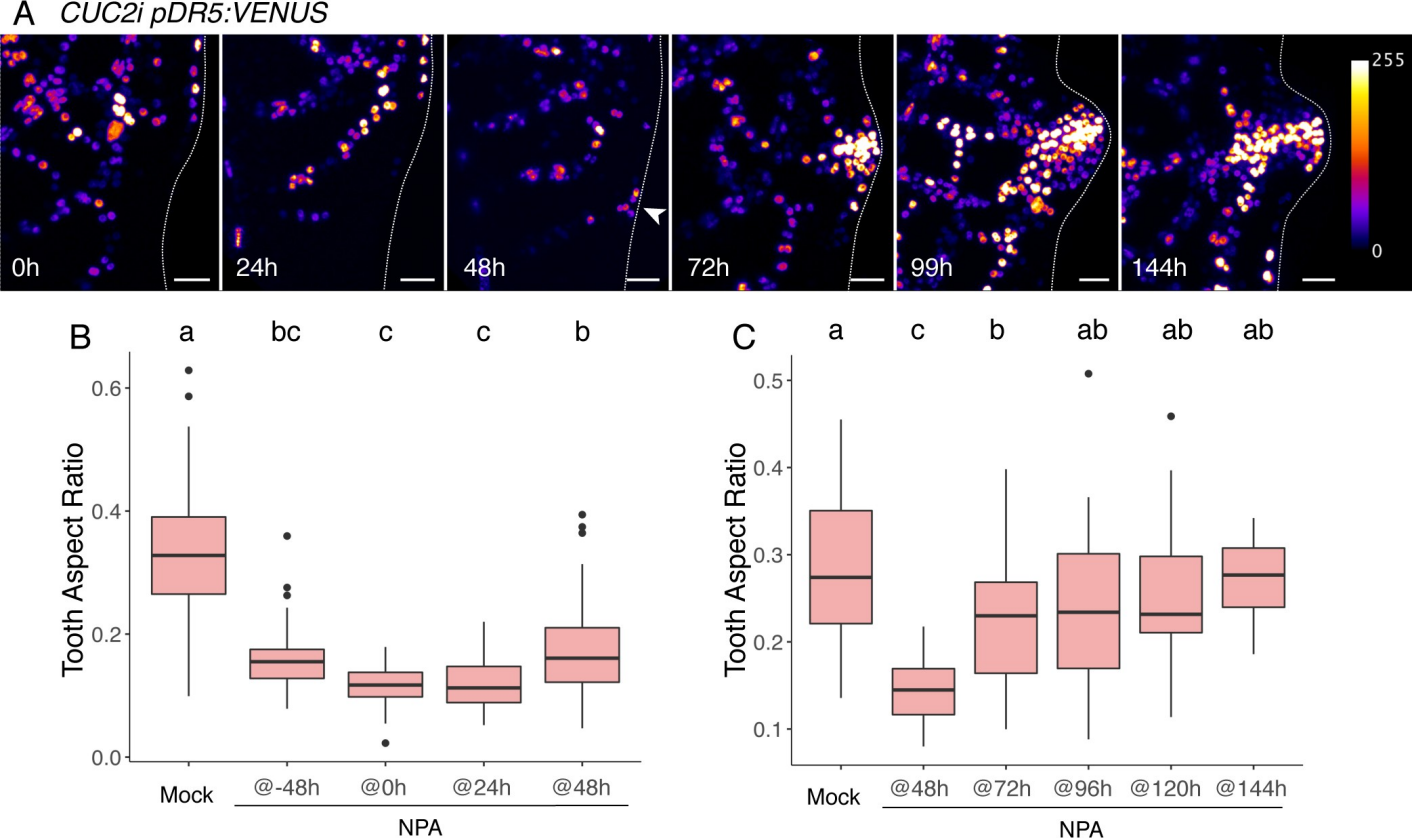
Auxin is a long-lasting functional relay for CUC2-triggered tooth outgrowth. (A) Dynamics of *pDR5*:*VENUS* in a *CUC2i* background after an 8h ethanol induction. Time following the start of induction is indicated. Note that the 0h time-point corresponds to an un-induced control. (B,C) Tooth shape 168h after an 8h ethanol *induction* in a *CUC2i* background following NPA applications. Onset of NPA application time is indicated relatively to induction start (e.g. @24h indicates that NPA is first applied 24h after induction start). Data are mean ± SEM, tooth number *n* ≥ 26. Letters show treatments with no significant difference (p-value<0.01) in one-way ANOVA followed by Tukey’s HSD. Scale bars: 20μm.

First, we monitored auxin response after *CUC2i* induction using *pRPS5a*:*DII-VENUS (DII-VENUS* [67]) and *pDR5*:*VENUS* [38], which respectively report early and late steps of auxin signaling (Fig 6A, S5A and S5B Fig). Clear localized auxin response could be detected at 48h (Fig 6A and S5A Fig). While the *pDR5*:*VENUS* positive domain was maintained until 144h, the domain revealed by the more dynamic *DII-VENUS* reporter tended to shrink at 127h, suggesting that the local auxin response started to decrease. Because the level of the *pDR5*:*VENUS* reporter increased in lines with higher CUC2 expression levels (S5C Fig), it suggests that CUC2 levels are translated into different auxin response intensities.

Next, to test the contribution of local increased auxin response to tooth growth at different stages of tooth development we used a pharmacological approach. 1-N-naphthylphtalamic acid (NPA), a polar auxin transport inhibitor, was sprayed on rosettes at different time points relative to ethanol induction (for instance, NPA@24h designates NPA treatments that start 24 hours after ethanol induction). Such treatments perturbed auxin response patterns shown by the *pDR5*:*VENUS* reporter as early as 3h following NPA application (S5D Fig). NPA application at any stage of tooth formation (from @24h to @96h) impacted localized auxin response as monitored by the *pDR5*:*VENUS* reporter (S5E–S5H Fig), but had no effect on overall leaf blade growth (S5I and S5J Fig). NPA application starting early relative to ethanol induction (from @-48h to @48h) leads to the most severe inhibition of tooth outgrowth (Fig 6B and S5K and S5M Fig). Conversely, a progressive release of tooth growth inhibition was observed when NPA was applied at later times (from @48h to @144h, Fig 6C and S5L and S5N Fig). Because in the case of late NPA applications (from @48h to @144h), localized auxin response could build up from 48h (Fig 6A and S5A Fig) to the time of NPA application, we concluded that localized auxin response continuously promotes tooth outgrowth and that it can therefore act as a long-lasting and quantitative functional relay contributing to tooth outgrowth after CUC2 becomes undetectable.

### *KLUH* also acts as a relay for CUC2-triggered tooth outgrowth

**Fig 7:**
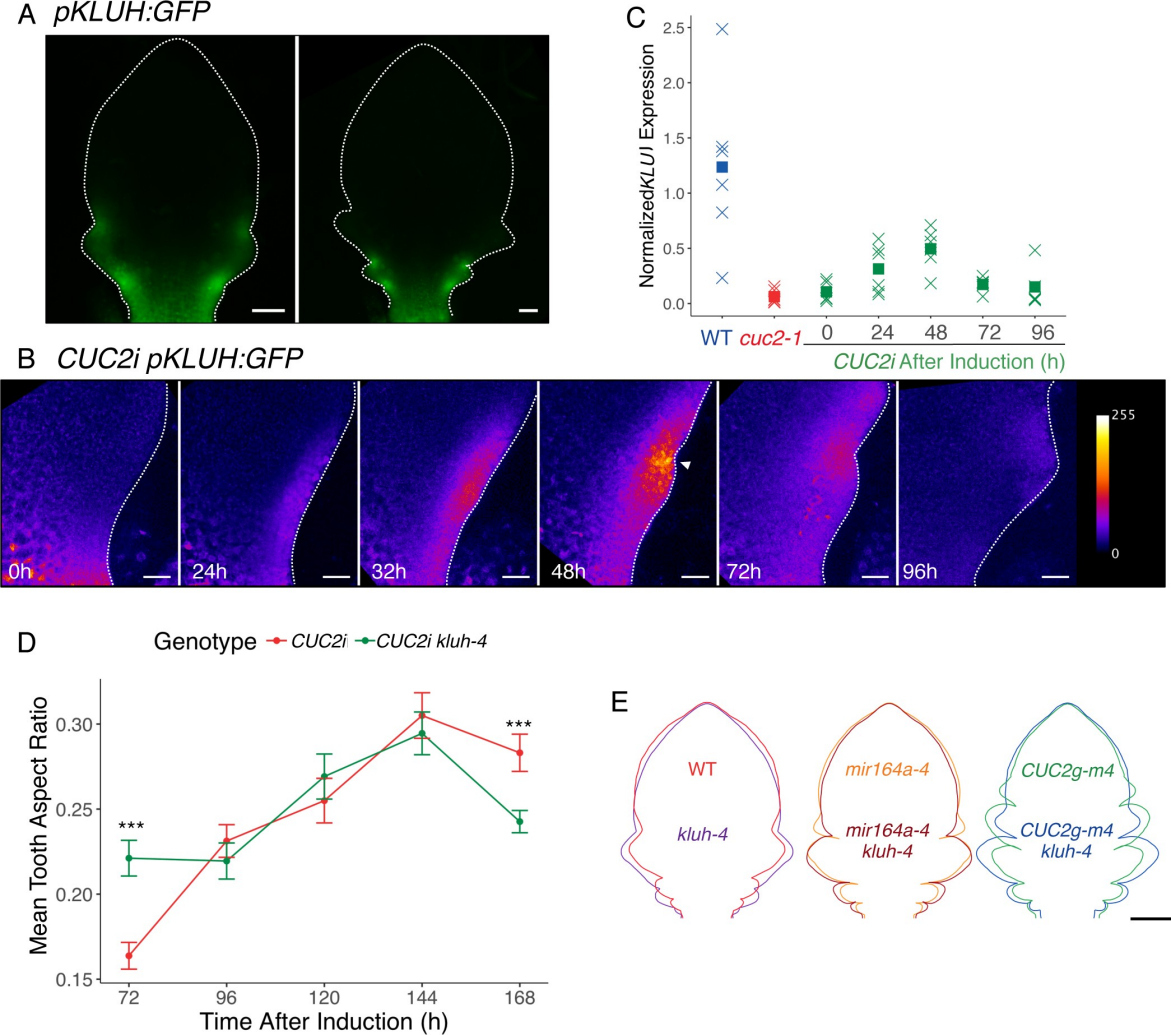
*KLUH* also acts as a functional relay for CUC2-triggered tooth outgrowth. (A) Expression of a *pKLUH*:*GFP* reporter during leaf development in a wild-type background. (B) Dynamics of *pKLUH*:*GFP* pattern in a *CUC2i* background after an 8h induction. Time following the start of induction is indicated. Note that the 0h time-point corresponds to an un-induced control. (C) Real-time RT-PCR quantifications of *KLUH* in the wild type (WT), *cuc2-1* and *CUC2i* at 0 to 96 hours after an 8h ethanol induction. Total RNAs were extracted from microdissected leaf margins and *KLUH* levels are normalized by *EF1α* and *qREF*. Crosses represent individual data points while squares are mean of the different samples, sample number *n* ≥ 5. (D) Tooth aspect ratio dynamics after an 8h ethanol induction in a *CUC2i* and *CUC2i kluh-4* background. Data are mean ± SEM, tooth number *n* ≥ 41. Statistical significance (Student’s test) is designated by * p<0.05, *** p<0.005. (E) Mean contours of 1100μm-long leaf 11, 12 and 13 WT/*kluh-4*; *CUC2g-m4*/*CUC2g-m4 kluh-4*; *mir164a-4*/*mir164a-4 kluh-4*. Scale bars: (A) 50μm, (B) 20μm, (E) 200μm.

To explore the existence of additional CUC2 relays, we reasoned that they should be expressed in a pattern similar to *CUC2*. Among the boundary enriched genes listed by [68], figured the *KLUH/CYP78A5* gene that was also previously described as expressed in meristem boundaries [69]. Interestingly, *KLUH* has been described as a non-cell autonomous regulator of cell proliferation in flowers and during seed development [60,62,70]. Although *KLUH* is expressed in the leaf [61], it is not known if it is expressed during serration formation. To test this, we followed the expression of a *pKLUH*:*GFP* reporter during wild-type leaf development (Fig 7A). In addition to the expression at the base of the petiole, *pKLUH*:*GFP* is also expressed in the sinuses of developing teeth. This prompted us to test whether *KLUH* is involved in the CUC2-triggered tooth formation process.

First, we monitored *KLUH* dynamics after *CUC2i* ethanol induction (Fig 7B). Interestingly, no *pKLUH*:*GFP* could be detected at the leaf margin before ethanol induction, confirming that *KLUH* expression is associated with boundary formation. Twenty-four hours after CUC2 induction, *pKLUH*:*GFP* was expressed at the leaf margin and became localized to the sinuses at 48h, overlapping with CUC2 (Fig 7B and S6A Fig). Then, *pKLUH*:*GFP* expression rapidly decreased, being almost undetectable at the leaf margin at 96h. Quantification of *KLUH* mRNA levels by RT-qPCR on microdissected leaf margins confirmed its transient upregulation following ethanol induction (Fig 7C).

Next, we tested the contribution of *KLUH* to CUC2-triggered tooth outgrowth by comparing tooth morphology in presence or absence of functional *KLUH* following CUC2 induction (Fig 7D and S6B Fig). Surprisingly, 72h after induction, teeth were pointier and the sinus angle more pronounced in *CUC2i kluh-4* compared to *CUC2i*. Later, morphological parameters became identical for teeth of both lines, while at 168h teeth were flatter with shallower sinus angles in the *CUC2i kluh-4* background compared to *CUC2i*. This complex effect of *kluh-4* on tooth growth was not limited to ethanol-induced tooth, as morphometric analysis of *kluh-4* revealed identical defects (Fig 5, blue compared to red). In small leaf primordia (blade <500μm) both teeth 1 and 2 of *kluh-4* were pointier compared to wild type, while in larger primordia (blade >1000μm) they were flatter in the *kluh-4* mutant. In addition, *KLUH* expression level correlates with CUC2 levels as RT-qPCR quantification showed that *KLUH* mRNA levels were increased in *CUC2g-m4* (S6C Fig). The *kluh-4* mutation also partially suppressed the increased leaf serration of *miR164a-4* and *CUC2g-m4* (Fig 7E and S6D Fig). We conclude from these results that CUC2 activates *KLUH* expression at the leaf margin. In turn, expression of *KLUH* has a dual role, transiently repressing tooth growth during the early stages of tooth formation while promoting it later.

**Fig 8:**
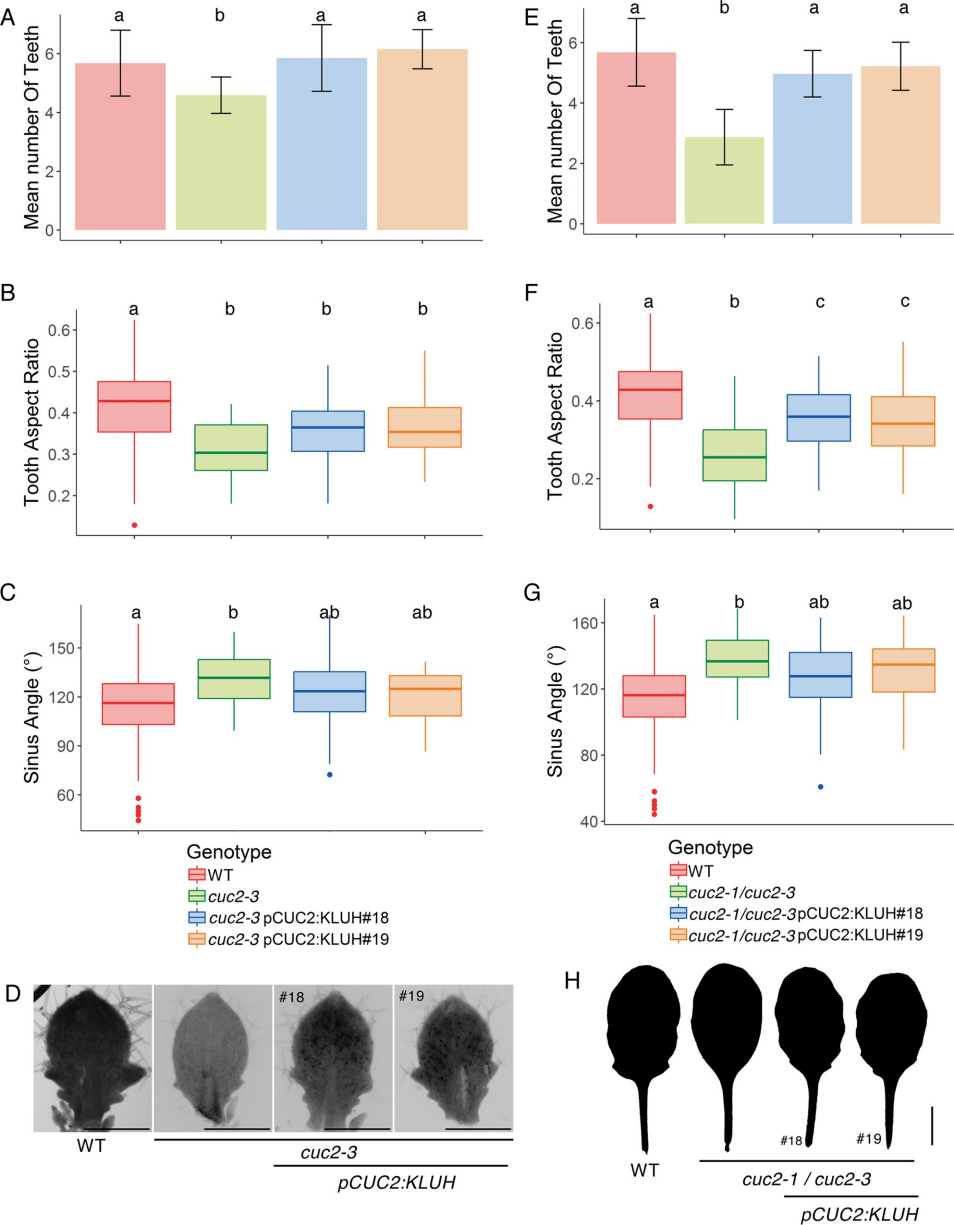
KLUH can partially compensate for CUC2 activity. (A-D) Morphometrics of the *pCUC2*:*KLUH* construct effects in the *cuc2-3* homozygous mutant or (E-H) transheterozygote *cuc2-1/cuc2-3*. The effects of two independent transformation events (#18 and #19) were characterized. *pCUC2*:*KLUH* was homozygous in the *cuc2-3* while it was hemizygous in the transheterozygote *cuc2-1/cuc2-3*. The leaf primordia were between 600 and 1500μm. (A,E) Mean tooth number ± SEM, (B,F) tooth aspect ratio and (C,G) sinus angle are shown. (D) Leaf primordia about 1mm-long of wild type, *cuc2-3* and two independent transgenic lines *cuc2-3 pCUC2*:*KLUH*. (H). Almost fully grown leaves of wild type, *cuc2-3* and two independent transgenic lines *cuc2-1*/*cuc2-3 pCUC2*:*KLUH*. Tooth number *n*≥19; aspect ratio and sinus angle *n*≥34. Letters show treatments with no significant difference (p-value<0.01) in one-way ANOVA followed by Tukey’s HSD. Scale bars = 500μm in (D) and 0.5cm in (H).

To further test whether *KLUH* conveys some of the growth promoting effect of CUC2, we expressed a *pCUC2*:*KLUH* construct in the *cuc2-3* background in which tooth growth is severely reduced (Fig 8A–8D). Tooth development was partially restored in *cuc2-3 pCUC2*:*KLUH* lines, as more teeth could be observed and as they were pointier and had more pronounced sinus. When *CUC2* gene dosage was further reduced in the *cuc2-1/cuc2-3* heterozygote a similar partial restoration was observed in small primordia (Fig 8E–8G) and extended to almost fully expanded leaves (Fig 8H). This indicates that expressing *KLUH* in the boundary can partially compensate for reduced *CUC2* activity during tooth formation and establishes *KLUH* as an important transiently-activated relay for CUC2-triggered tooth formation.

### Interactions within the network regulating morphogenesis at the leaf margin

**Fig 9:**
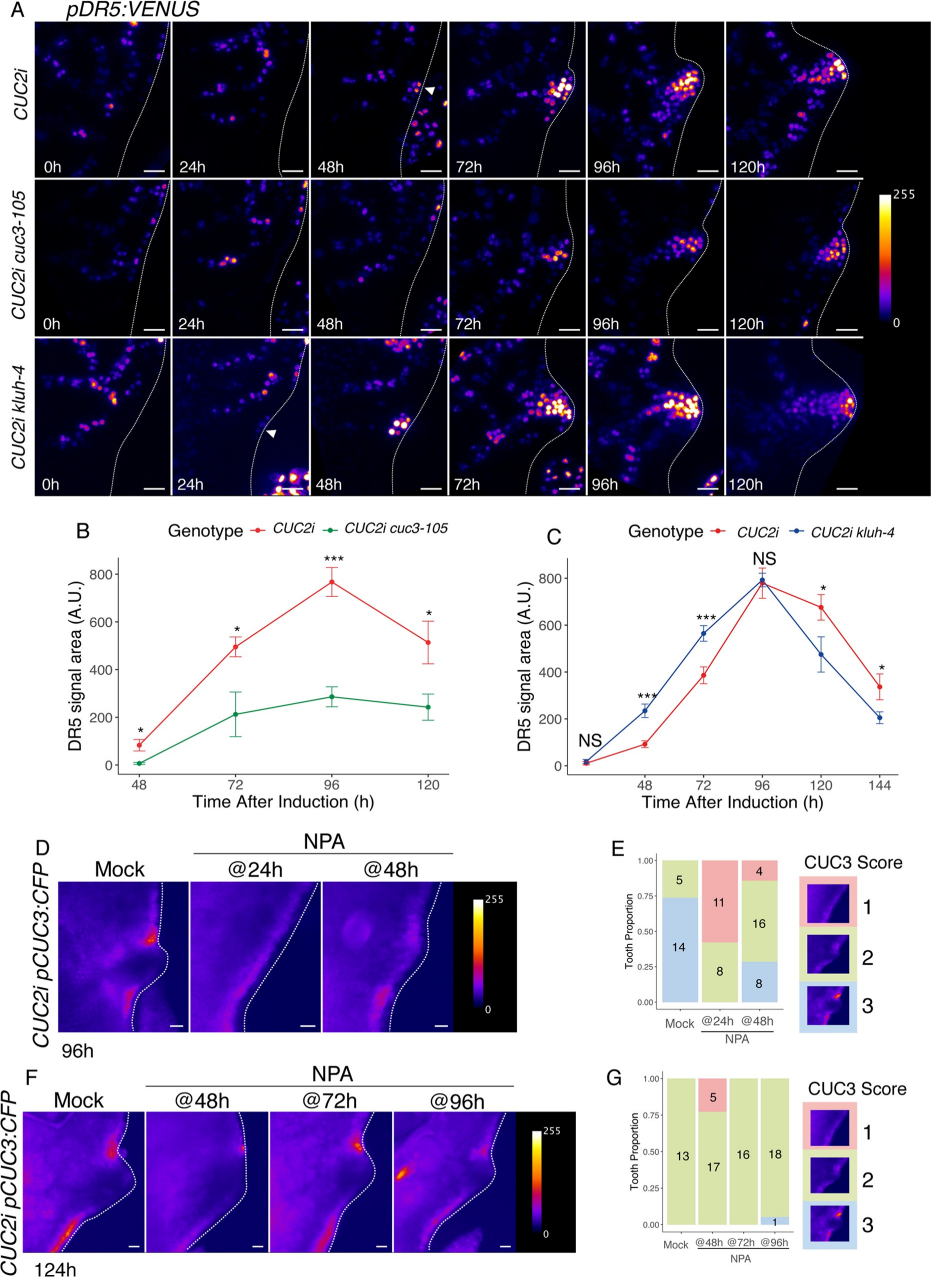
Interactions between CUC2 relays during tooth formation. (A) Dynamics of *pDR5*:*VENUS* after an 8h ethanol induction in either *CUC2i*, *CUC2i cuc3-105* or *CUC2i kluh-4* backgrounds. Time following induction start is indicated. The white arrowhead points to the newly formed *pDR5*:*VENUS* maximum at the leaf margin at 48h in the *CUC2i* background, while at the same time no such maximum is visible in the *CUC2i cuc3-105* background. Note that the pDR5:VENUS maximum domain remains smaller and less strong in the *CUC2i cuc3-105* background compared to the control *CUC2i* line. Note that the 0h time-point corresponds to an un-induced control. (B-C) DR5-VENUS signal area quantification in the *CUC2i cuc3-105* (B) or *CUC2i kluh-4* (C) backgrounds compared to CUC2i, along time after induction. DR5-VENUS signal area was quantified on images after applying a threshold that allows to separate signal from background. Data are mean ± SEM, tooth number *n* ≥ 4. Statistical significance (Student’s test) is designated by * p<0.05, *** p<0.005. (D) *pCUC3*:*CFP* expression observed 96h after an 8h ethanol induction in a *CUC2i* background and following either NPA application 24h (NPA @24h) or 48h (NPA @48h) after ethanol induction start. Pixel intensity is represented with the Fire LUT (E) Scoring of discontinuity in *pCUC3*:*CFP* expression in a *CUC2i* background after an 8h ethanol induction and following NPA applications. The classes used to score *pCUC3*:*CFP* expression were defined based on the dynamics observed for its expression following induction of RFP-CUC2 expression (see Fig 4B): class 1 continuous expression profile; class 2 discontinuous profile, class 3 discontinuous profile and strong signal in the margin epidermis. Sample number is indicated in bars. (F) *pCUC3*:*CFP* expression observed 124h after an 8h ethanol induction in a *CUC2i* background and following either NPA application 48h (NPA @48h), 72h (NPA @72h) or 96h (NPA @96h) after ethanol induction start. Pixel intensity is represented with the Fire LUT. (G) Scoring of discontinuity in *pCUC3*:*CFP* expression in a *CUC2i* background after an 8h ethanol induction and following late NPA applications. Scoring was done as in (E) except that the observations were made 124h after the start of the ethanol induction instead of 96h. Scale bars: (A) 20μm; (D, F) 50μm.

We showed above that CUC2 expression at the future sinus sites induces locally the expression of two boundary genes, *CUC3* and *KLUH*, and leads to strong auxin response at a distance. Because tooth morphogenesis requires a coordination between growth repression at the sinus and growth promotion at the tip, we next tested the interactions between factors acting in the sinus and acting at the tip of the tooth. To test whether *CUC3* and *KLUH* also contribute to localized auxin response, we monitored *pDR5*:*VENUS* after induction in *CUC2i*, *CUC2i cuc3-105* and *CUC2i kluh-4* backgrounds (Fig 9A, 9B and 9C). *pDR5*:*VENUS* upregulation at the leaf margin appeared with a 24 hours delay in *CUC2i cuc3-105* compared to *CUC2i*, and the area of cells expressing the reporter and its expression level remained lower than in *CUC2i* until 120h (Fig 9A and 9B). This indicates that *CUC3* contributes, along with CUC2, to properly set the dynamic and intensity of the local auxin response maximum. Monitoring *pDR5*:*VENUS* expression in *CUC2i kluh-4* revealed a complex modification compared to *CUC2i* (Fig 9A and 9C): *pDR5*:*VENUS* expression was detected earlier in *CUC2i kluh-4* at 24h compared to *CUC2i* and remained stronger until 96h when it became similar in *CUC2i* and *CUC2i kluh-4*. Later at 120h and 144h, *pDR5*:*VENUS* expression was weaker in the *kluh-4* background. This indicates that the changing effects of *KLUH* on tooth outgrowth depending on the developmental stages (Fig 7D) are correlated with similar effects on the auxin response visualized by *pDR5*:*VENUS*.

Next, we conversely tested the contribution of localized auxin response to the dynamics of *CUC3* and *KLUH* patterns. When NPA was applied early following ethanol induction (@24h or @48h) *pCUC3*:*CFP* expression remained low and continuous along the margin (Fig 9D and 9E). This indicates that *pCUC3*:*CFP* pattern requires localized auxin response upregulation to become discontinuous as does RFP-CUC2 (S7A Fig). But interestingly, later NPA applications did not impact *pCUC3*:*CFP* discontinuous pattern (Fig 9F and 9G), suggesting that although local auxin response is important to initially restrict *CUC3* expression to the sinuses, this pattern is later maintained in an auxin and CUC2-independent manner. Because *pKLUH*:*GFP* is only expressed at early stages (Fig 7B), we could only test the effects of early NPA application @24h. In contrast to *pCUC3*:*CFP*, *pKLUH*:*GFP* pattern 48h after induction was not modified by early NPA applications (@24h; S7B Fig) indicating that local auxin response is not required for *KLUH* expression to arise at the leaf margin.

Altogether, this shows that CUC2-mediated induction of *CUC3* and *KLUH* expression is required for proper dynamics of the auxin response at the leaf margin, and that in turn strong auxin response is necessary for the initial establishment of *CUC3* expression pattern, but not for its later maintenance.

## Discussion

### A model for patterning and growth coordination by plant boundaries

In plants, the repeated formation of new growth axes throughout their lives is the basis for their developmental plasticity, permitting adaptation to the environment. In the shoot and flowers, formation of such new growth axes relies on the interplay between local upregulation of auxin response, that determines the position of the new growth axis, and a boundary domain that contributes to its individualization [24,25,30,56,71,72]. Until now, patterning and growth during the formation of new axes appeared intimately intertwined as they rely on the same regulators, auxin and the *CUC* genes. Here, using tooth formation at the leaf margin as a model, we show that patterning and growth can be genetically separated, as the former strictly requires CUC2 while the latter can happen independently of CUC2. Nevertheless, we provide evidence that CUC2 acts as a quantitative trigger for growth, as CUC2 levels direct growth rate through the quantitative activation of three downstream functional relays, *CUC3*, *KLUH* and auxin response. Although these relays all contribute to growth, our functional analyses show that they act at different points in time and space. In particular, *CUC3* acts locally while auxin has a most distant and very long-lasting role for sustained growth. We also reveal the involvement of a new actor, *KLUH/CYP78A5*, during leaf margin morphogenesis, showing that it can partially substitute for *CUC2* to promote tooth outgrowth.

**Fig 10:**
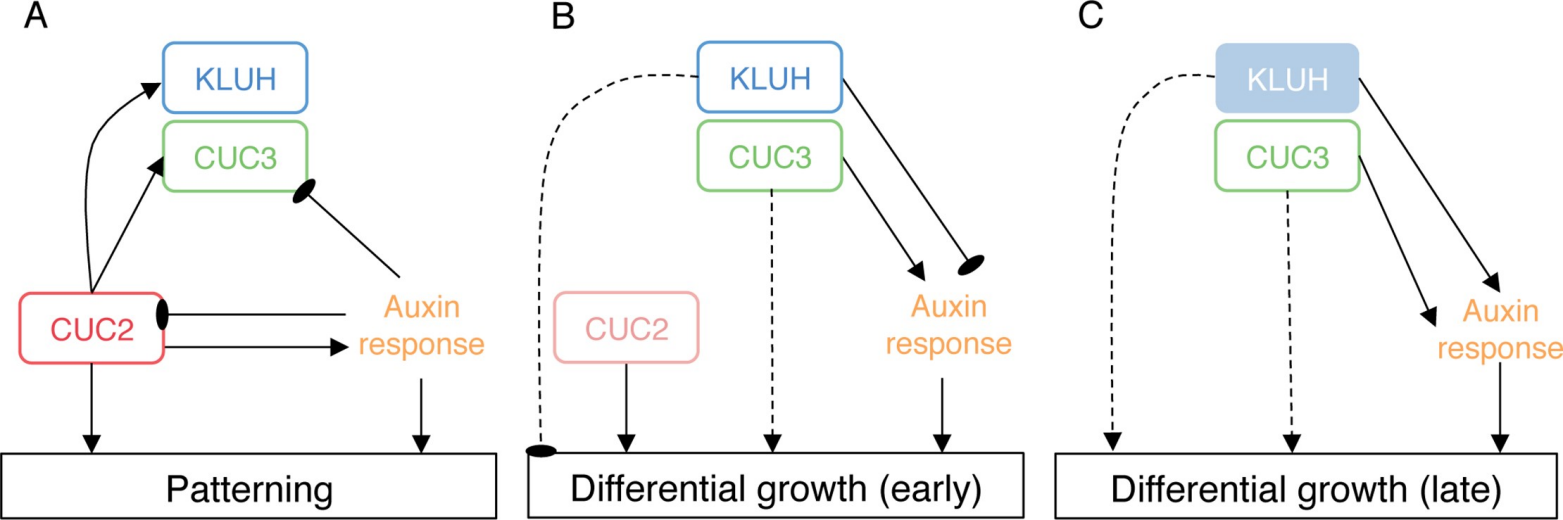
Dynamic model for CUC2-induced boundaries patterning and growth during tooth morphogenesis. (A) CUC2 sets leaf margin patterning by: (i) modifying *CUC3* expression pattern (ii) activating *KLUH* expression (iii) and creating auxin transport polarity convergence points through a cell-autonomous mechanism that builds-up high auxin response foci. In turn, high auxin response spatially restricts *CUC2* and *CUC3* expression. (B) Once the pattern is established, CUC2 becomes dispensable for tooth outgrowth (light pink). *KLUH* and *CUC3* are expressed in the sinus and they both modulate auxin response that is the main driver for tooth outgrowth. (C) In a later phase, *KLUH* is not expressed anymore. However, *KLUH* promotes auxin response either indirectly or by the production of a persistant signal (light blue). *CUC3* promotes auxin response. Whether *KLUH* and *CUC3* control growth events independently of auxin is still unknown (dashed line).

Based on our observations we propose a three-step mechanism for tooth morphogenesis (Fig 10). During the first phase, the leaf margin is patterned into a boundary domain marked by *CUC2*, *CUC3* and *KLUH* expressions and a tooth tip domain characterized by a high auxin response. Such a patterning is initiated by CUC2 that promotes *CUC3* and *KLUH* expressions and leads to the formation of a strong auxin response at distance via modification of PIN1-mediated auxin transport [55,56]. In turn, auxin response contributes to refine *CUC2* and *CUC3* expression patterns. During the second phase for which CUC2 is not absolutely required, differential growth is initiated as a result of a strong auxin response that is promoted by *CUC3* and inhibited by *KLUH*. Expression of *CUC3* may also contribute to differential growth by locally repressing growth in the boundary domain as was suggested for CUC2 [65]. During the third phase, although *KLUH* is likely not expressed anymore, its effect remains and maintains auxin response and growth high. Overall, this model provides a scenario by which the boundary domains coordinate patterning and growth events during the formation of new growth axes

### *CUC2* orchestrates an interconnected network that can promote growth and may contribute to robust developmental responses

Our results reveal the central role of CUC2 in the activation of an interconnected downstream network that promotes growth. Our quantifications show that variations in CUC2 levels are translated into different activity levels of the downstream actors, in particular auxin response that appears to control growth quantitatively. Another important feature of the network is that, although it initially enlarges downstream of CUC2 into three downstream actors, it seems to converge on auxin response. CUC2 promotes the formation of a strong auxin response at distance. In parallel, our observations suggest that CUC2 sets the expression of *CUC3* and *KLUH* at the leaf margin. In addition, auxin response and also other factors may contribute to their expression dynamics. For instance, because *CUC3* expression was shown to be regulated by mechanical stresses in the meristem [41], one could imagine that similar mechanical stresses accompany tooth outgrowth and contribute to *CUC3* local upregulation.

Our pulsed CUC2 expression experiments show that this network can substitute for CUC2 to promote growth. However, during wild-type leaf development CUC2 expression is maintained during a longer duration and temporally overlaps with those of the 3 functional relays we identified. Furthermore, our results show strong interconnections between the actors of the network as both *CUC3* and *KLUH* contribute to modulate auxin response and, that conversely, a strong auxin response is required for the proper dynamics of *CUC3* and *CUC2* (our results and [55,56]). Such an interconnected network may allow buffering stochastic variations in the activity of individual actors of the network to provide a robust developmental response while providing enough flexibility to transduce stable variations of their activity into quantitative differences in growth, as we show here for CUC2.

### *KLUH* controls leaf morphogenesis, possibly via auxin response modulation

We show that *KLUH* expression at the leaf margin is activated in response to CUC2. In addition, proper tooth growth, either following ethanol-induced restoration of CUC2 expression or in wild-type leaf margins requires *KLUH*, suggesting that part of CUC2 effects on growth occurs via *KLUH*. Indeed, we show that tooth growth is partially restored in *cuc2-3* and *cuc2-1/cuc2-3* when a *pCUC2*:*KLUH* construct is introduced, demonstrating that *KLUH* is an important player in the CUC2-induced network promoting tooth growth. *KLUH* has been previously described as controlling final organ size by regulating cell proliferation timing in a non-cell-autonomous mechanism [60,61,70]. Interestingly, the unknown mobile signal hypothetically produced by KLUH was suggested to be able to control growth of adjacent organs in flowers and even different flowers in the inflorescence, indicating that it could diffuse on long distances (on the cm range) [60,62]. But *KLUH*’s involvement on leaf margin morphogenesis implies much shorter diffusion ranges for the mobile signal (on the range of tooth size, which is about a few hundred μm). This suggests that the mobile signal could have different diffusion ranges depending on the organ or the developmental stage.

Initial studies suggested that *KLUH* acts independently of classical phytohormones [60]. However, overexpression of *PLASTOCRHON1*, a gene that belongs to the same class of *CYP78A* as *KLUH* (although it is located in a different clade), has been recently shown to lead to higher auxin levels and response during maize leaf development [73]. Our work supports a role for *KLUH* in the modulation of auxin response, which appears to strictly correlate with the dynamic effects of *KLUH* on growth. However, whether all the *KLUH* effects on growth are mediated by changes in auxin response or whether *KLUH* acts on growth also independently of auxin signaling remains to be determined.

Another standing question is the basis of the bimodal action of *KLUH* shown here, as *KLUH* represses early tooth growth while promoting it at later stages. One possibility is that cellular responses to *KLUH* may vary in time or space, thus providing the basis of the contrasted effects of *KLUH*. Alternatively, the latter effect could be an indirect consequence of the early effect. For instance, early modification of auxin transport or signaling could affect these processes during later stages. Interestingly, such a bimodal action also occurs at the whole plant scale, as leaf primordia arise faster but show reduced growth in the *kluh* mutant [60,74], indicating that it is not specific to morphogenesis at the leaf margin.

In conclusion, we show that plant boundaries coordinate patterning and growth to direct morphogenesis and we provide evidence that these two processes can be temporally and genetically separated, as the former requires CUC2 while the latter can occur independently of CUC2. In the absence of CUC2, differential growth is maintained via the activation of a regulatory network that can act as a functional relay. Like animal boundaries, plant boundaries control morphogenesis through multiple pathways, but they differ in their effect on morphogen distribution. While in animals, morphogens are produced by the boundary and therefore form a boundary–centered gradient [2,4], plant boundaries locally modify the distribution of the morphogenetic regulator auxin and lead to its accumulation at distance. In addition, while animal morphogens have been proposed to control proliferation in a concentration-independent manner [75,76] our results indicate that auxin controls growth in a quantitative manner. However, we show that auxin, like the morphogen Decapentaplegic, is continuously required throughout development to promote growth [77–79]. Beside auxin, the putative mobile signal produced by *KLUH* in the boundary domain could form a morphogen-like gradient, similar to animal morphogens. Validation of such a hypothesis awaits the identification of the putative signal.

## Materials & methods

### Plant material and growth conditions

All genotypes are in the Columbia-0 (Col-0) ecotype. The *cuc2-1* mutant was originally isolated in the Landsberg *erecta* ecotype but was backcrossed 5 times in Col-0 [48]. The *mir164a-4* [29], *cuc2-3*, *cuc3-105* [23], *ago1-27* [80], *kluh-4* [60], and *jaw-D* [81] mutants were previously described, as well as the *pCUC3*:*CFP* [26], *pCUC2*: RFP [82], pDR5:*VENUS* [38] *pRPS5a*:*DII-VENUS*, *pRPS5a*:*mDII-VENUS* [67] and *CUC2g-m4* [29] transgenic lines. The *cuc2-3 pCUC2*:*CUC2*:*VENUS* line (33) used was described before [26] and two additional lines (44 and 45) were generated using the same approach in the *cuc2-1* background. Heterozygous versions of the *pCUC2*:*CUC2-VENUS* reporter used in Fig 3 and S2 Fig were generated by crossing T3 homozygous plants bearing unique insertions of the reporters to homozygous *cuc2-3* or *cuc2-1* mutants.

Seeds were soaked in water at 4°C for 48 hours prior to sowing. Plants were grown in soil in short-day conditions [1 h dawn (19°C, 65% hygrometry, 80 μmol.m^-2^.s^-1^ light), 6 h day (21°C, 65% hygrometry, 120 μmol.m^-2^.s^-1^ light), 1 h dusk (20°C, 65% hygrometry, 80 μmol.m^-2^.s^-1^ light), 16 h dark (18°C, 65% hygrometry, no light)]. Plants from in S3C Fig were grown in vitro (Arabidopsis medium Duchefa) in long day conditions [16h light / 8h dark at 21°C].

### Molecular cloning and plant transformation

The *CUC2i* line was generated in several steps. First, we generated a *pCUC2*:*ALCR pAlcA*:*GUS* driver construct using a 3.7kb CUC2 promoter sequence used in a previously described *pCUC2*:*GUS* reporter [29] and transformed it into wild-type Col-0 background. Two lines with a single *pCUC2*:*ALCR pAlcA*:*GUS* locus site based on the segregation of hygromycin resistant and sensitive plants in the T2 generation and showing the expected GUS staining in the meristem and leaves upon ethanol induction were selected. These two lines were crossed with the *cuc2-1* mutant introgressed into Col-0 [48] and lines double homozygous for *cuc2-1* and *pCUC2*:*ALCR pAlcA*:*GUS* were identified in the resulting F3 generation. Second, we generated N- and C- terminal fusions of CUC2 with the RFP using pH7RWG2 and pH7WGR2 vectors [83]. The resulting fusions were cloned between the pAlcA and 35S terminator and inserted into a pGreen0229 and transformed into the *cuc2-1* mutant introgressed into Col-0. Third, at least fifteen primary transformants with either CUC2 fusion were crossed with the two *cuc2-1 pCUC2*:*ALCR pAlcA*:*GUS* double homozygous lines. Leaf serration rescue was observed following ethanol induction of the resulting F1 lines. We noticed no difference in the leaf serration rescue by the two fusions. To further confirm this we constructed two lines homozygous for *cuc2-1*, *pCUC2*:*ALCR pAlcA*:*GUS* and either *pAlcA*:*RFP-CUC2* or *pAlcA*:*CUC2-RFP*. Both lines responded similarly to varying durations of ethanol treatment (S2F and S2G Fig) and showed no detectable fluorescence in the cytoplasm (S2H Fig), suggesting that fusion with the RFP did not severely affect CUC2 function and was not cleaved off. We selected the RFP-CUC2 fusion for further analysis. Fourth, we generated 3 lines homozygous for *cuc2-1*, *pCUC2*:*ALCR pAlcA*:*GUS* and *pAlcA*:*RFP-CUC2* (with 2 independent transformation events for *pCUC2*:*ALCR pAlcA*:*GUS* and *pAlcA*:*RFP-CUC2*). We next characterized tooth formation in these lines following increasing levels of ethanol induction by watering the plants with 0.01% to 1% ethanol solutions. As we observed no difference between these lines we selected one of them, called *CUC2i* here, for detailed analyses.

For the *pMIR164A*:*RFP* reporter, the endoplasmic reticulum targeted RFP cassette from the *pCUC2*:*RFP* reporter [82] was cloned behind a 2.1 kb long *MIR164A* promoter [29] within a pGreen0129 vector. A line segregating a single locus based on the hygromycin resistance segregation and showing an expression pattern similar to the previously *pMIR164A*:*GUS* line was selected.

For the *pKLUH-GFP* reporter, a 4116 bp promoter sequence ending 10 bp after the initiation codon was amplified from Col-0 genomic DNA and cloned in front of a GFP-NOS terminator cassette contained in a pGreen0229 vector. This 4.1 kb 5’genomic region was shown to be sufficient to rescue a *kluh-2* mutant when driving a KLUH–vYFP fusion [60]. Fifteen primary transformants were identified based on their resistance to basta and we selected one that showed the expected expression pattern at the organ basis and integrated the *pKLUH-GFP* construct at a single locus based on the segregation of basta resistant and sensitive plants in the T2 generation.

For the *pCUC2*:*KLUH* construct, the full *KLUH* coding sequence was amplified from Col-0 seedling cDNA, cloned into the pGEM-T Easy System Vector and sequenced. The KLUH CDS was cloned as a NotI fragment into a pGreen0129-t35S-Pro_CUC2_ vector [48] to generate the *pCUC2*:*KLUH* construct. The resulting construct was sequence-verified and transferred into *Agrobacterium tumefaciens* strain GV3101, and *cuc2-3* plants were transformed by floral dipping. Primary transformants were selected *in vitro* for their resistance to hygromycin. Two independent lines were selected based on a hygromycin resistance segregation indicating the integration of the transgene as a single locus. pCUC2:KLU#18.1 and #19.6 homozygous lines were used for further analyses. Transgenic lines were genotyped for the presence of both the *cuc2-3* mutation and the *pCUC2*:*KLUH* transgene. The *cuc2-1/cuc2-3 pCUC2*:*KLUH* transheterozygotes were constructed by crossing the homozygous *cuc2-3 pCUC2*:*KLUH* with *cuc2-1*.

### Leaf sample preparation prior to imaging

Plants were grown for 3 to 4 weeks prior to observations. All observations are done in leaves with a rank higher than 10, which were imaged with their adaxial face closer to the objective. Typically 10–20 leaves coming from 3–6 different plants were imaged. Leaves were isolated from the meristem using surgical syringe needles and mounted between slide and coverslip. Mounting media has the following composition: Tris HCl 10mM pH = 8,5, Triton 0,01%.

### Imaging

Confocal imaging (Figs 2E, 4B, 6A, 7B and 9A and S2H, S4A, S5A, S5B,S5D, S6A and S7A Figs) was performed on a Leica SP5 inverted microscope (Leica Microsystems, Wetzlar, Germany). Lenses are Leica 20x or 40x HCX PL APO CS. Acquisition parameters are presented in S1 Table and were kept constant throughout acquisitions so that intensity levels are comparable.

The binocular imaging (Figs 1A,1C, 2B, 4A, 7A, 8D, 9D and 9F and S2A, S5E,S5G,S5M,S5N and S7B Figs) was done using an Axio Zoom.V16 macroscope (Carl Zeiss Microscopy, Jena, Germany, http://www.zeiss.com/), RFP was imaged using a custom-made filter block (excitation band pass filter 560/25; beam spliter 585, emission band pass filter 615/24, AHF, Tuebingen, Germany, https://www.ahf.de/), CFP was imaged using the Zeiss 47 HE filter set (excitation band pass filter 436/25; beam spliter 455, emission band pass filter 480/40), VENUS was imaged using the Zeiss 46 HE filter set (excitation band pass filter 500/25; beam spliter 515, emission band pass filter 535/30), and fluorescence of the chlorophyll was imaged using the Zeiss 63 HE filter set (excitation band pass filter 572/25; beam spliter 515, emission band pass filter 535/30).

Figures were made using the ImageJ plugin FigureJ [84]. Most images are represented using the Fire LUT from ImageJ. In this case the Fire LUT was applied to the whole panel after it was assembled and a calibration bar is provided on the panel’s right end. White dashed lines always mark the leaf margin limit.

### Image based CUC2 quantity evaluation

We quantified RFP-CUC2 and CUC2-VENUS fluorescence in the CUC2 domain along the margin of young leaf primordia on confocal images obtained as described above. After tooth initiation that leads to discontinuous CUC2 domains [56], we focused on the sinus distal to the first tooth, as *CUC* expression in this domain has been shown to drive the outgrowth of marginal structures [30,66]. The quantification of the RFP-CUC2 fluorescence was manually performed using ImageJ. The intensity of the 12 most intense nuclei was measured on the medial plane of each nucleus. The mean intensity of the background was substracted from the mean of the intensity of the nuclei. The pCUC2:CUC2-VENUS signal was quantified by a similar approach ([82] for details).

### Image based promoter activity quantification

*pCUC2*:*RFP*, *pCUC3*:*CFP* and *pDR5*:*VENUS* signal quantifications presented in S3C and S3D Fig and S4C Fig were performed on Axio Zoom.V16 macroscope obtained images (see above) using the Qpixie macro previously described [82].

### Leaf shape phenotyping

Most measures were manually performed using ImageJ on pictures made either with the binocular or the confocal microscope. *Blade Length* is defined as the length between the blade petiole junction and the leaf apex. *Tooth With* is the distance between two consecutive primary sinuses. *Tooth Height* is the tooth altitude, which is the distance starting at the tooth tip and meeting perpendicularly the tooth width segment. In order to evaluate the anisotropic tooth growth without taking global leaf growth into account, we normalize *Tooth Height* by *Tooth Width* and call this new parameter *Tooth aspect ratio*. The *Sinus Angle* is the local angle formed by the blade margin at the distal sinus site (see S2D Fig).

Phenotype quantifications and mean leaf contours in Figs 4E and 7E, S3E and S5D Figs were performed using the Morpholeaf application installed on the FreeD software [65,85].

Dissection index (DI) presented in S3E and S5D Fig is defined as $DI=\frac{{Leaf\;perimeter}^{2}}{4.\pi .Leaf\;area}$. A perfect circle has DI = 1.

### Pharmacological treatments

Ethanol inductions were performed on 3-week-olds plants using ethanol vapors for the time indicated in the figure legends. Plants were covered with plastic covers during induction.

NPA way sprayed on plants until they were covered in solution. Spraying solution: NPA 10μM, DMSO 0,1%, Triton 0.01%. Once NPA applications were started, plants were sprayed every two days. In order to have identical total number of spray applications between the different treatments, plants were mock treated on days they did not receive NPA.

### Laser assisted microdissection and RNA extraction

Leaf margins were microdissected with the ZEISS PALM MicroBeam using the Fluar 5x/0.25 M27 objective. Leaves under 2mm long of rank >10 were placed on MMI membrane slides (Prod. No. 50103) and microdissected samples were collected in ZEISS AdhesiveCaps. Cutting parameters are the following: speed 10–15%, energy: 67%, focus: 76%. Approximately 20 microdissected leaf margins were collected in each sample. Total RNAs were extracted using the Arcturus PicoPure RNA Isolation Kit following manufacturer’s instruction. RNA quality was controlled using the Agilent RNA 6000 Pico Kit.

### RNA extraction on whole seedlings

Total RNA were isolated using using RNAeasy Plant Mini Kit (Qiagen) following manufacturer’s instruction for plant tissue including on-column DNAse treatment. Reverse transcription was performed using RevertAid H Minus M-MuLV Reverse transcriptase (Fermentas) using 2μg of total RNA.

### Real-time PCR expression analysis

Real time PCR analysis was performed on a Bio-Rad CFX connect machine using the SsoAdvance Universal SYBR Green Supermix following manufacturer’s instruction. PCR conditions are as follows: Conditions: 95°C 3min; (95°C 10s; 63°C 10s; 72°C 10s) x45 cycles. Primers used for real time PCR analysis are available in S2 Table. Analysis was carried out using the ΔΔCt method [86].

### Statistical analysis

Statistical analysis were performed on R [87] and graphical output was produced with the package ggplot2.

## Supporting information

S1 Fig CUC2 mRNA level quantification in wild type, *cuc2-1* and *cuc2-3*. Real-time RT-PCR quantifications of *CUC2* mRNA levels in the wild type (WT), *cuc2-1* and *cuc2-3* mutants. Total RNAs were extracted from 2 week-old plants dissected to remove all leaves and *CUC2* mRNA levels were normalized by *EF1α* and *qREF*. Each point represents a biological replicate.
(PDF)

S2 Fig *CUC2i* line characterization, related to Fig 2. (A) Prolonged *CUC2i* induction restores multiple teeth formation. Leaf silhouettes from leaves of comparable rank from wild type (WT), *cuc2-1* and *CUC2i* plants induced by ethanol for 5x8h and observed 9 days after induction.
(B) Only small leaf primordia (< ∼1200μm) form teeth following *CUC2i* induction. The primordia size of leaves L10 to L14 was measured at the induction start in half of the plants. The number of teeth formed following a 6h ethanol induction on L10 to L14 was determined one week after induction on the other half of the plants. Data are mean ± SD, leaf number *n* ≥ 8.
(C) *CUC2i* induction duration determines the number of teeth formed. Data represent mean ± SEM (leaf number *n* = 12) of teeth number formed following 1h to 48h ethanol inductions. Teeth were counted one week after the induction start on the three most dissected leaves.
(D) Representation of the *Tooth Aspect Ratio* that is defined as the tooth height (h) / tooth width (w) ratio. It quantifies anisotropic growth and integrates both growth promotion at the tip and growth repression at the sinus. Representation of the *Sinus Angle* (α) measured in the distal sinus of the first tooth. It is a local parameter more directly related to the local growth repression in the sinus.
(E) *CUC2* mRNA is detected for two days following an 8h ethanol induction. Real-time RT-PCR quantifications of *CUC2* expression in the WT, *cuc2-1* and *CUC2i* at 0 to 96 hours after an 8h ethanol induction. RNAs were extracted from microdissected leaf margins and *CUC2* levels are normalized by *EF1*α and *qREF*. Crosses represent individual data points while squares are mean of the different samples, sample number *n* ≥ 2.
(F-G) The *CUC2i* line expressing a RFP-CUC2 fusion shows a response to varying durations of ethanol induction similar to that of a line expressing a CUC2:RFP fusion. (F) Data are mean ± SEM (leaf number *n* = 10) of teeth number formed following 2h to 48h ethanol inductions and (G) the mean ± SEM of the number of leaves showing at least one tooth. Observations were made one week after the induction start on the three most dissected leaves.
(H) Both RFP-CUC2 and CUC2-RFP fusions show RFP fluorescence localized to the nucleus, with no signal above background visible in the cytoplasm. Images were taken following 48h induction and a pixel intensity histogram along a segment is shown below.
Scale bar: (A) 500μm.
(PDF)

S3 Fig Details of the methods used to compare CUC2 levels with tooth morphogenesis related to Fig 3. (A,C) Tooth height evolution along blade length in different genotypes. Data are individual measures and a linear regression for each genotype is shown (for all genotypes r > 0.93). The regression slope is the Tooth growth rate in Fig 3.
(B,D) Quantification of CUC2-VENUS fluorescence and local regression during leaf development, each point is the mean ± SD of *n* = 12 nuclei per sinus. The grey area limits the interval used to calculate mean CUC2 quantity in Fig 3.
(PDF)

S4 Fig Detailed characterization of *CUC3* as a local functional relay for CUC2-triggered toth outgrowth, related to Fig 4. (A) Relative localization of RFP-CUC2 protein and expression of a *pCUC3*:*CFP* reporter after an 8h ethanol induction. The time following the start of induction is shown on the overlay panels. Note coexpression of RFP-CUC2 and *pCUC3*:*CFP* in the epidermis (arrowheads in A).
(B) Sinus angle dynamics after an 8h ethanol induction in a *CUC2i* and *CUC2i cuc3-105* background. Data are mean ± SEM (sinus number *n* ≥ 10). Statistical significance (Student’s test) is designated by * p<0.05, *** p<0.005.
(C) Correlation between *pCUC2*:*RFP* and *pCUC3*:*CFP* promoter activity in a wild-type background. The promoter activity is evaluated by quantifying fluorescence levels in developing first teeth for blade length <1000 μm. Data are represented as individual measures and a linear regression (r = 0.899).
(D) Quantification of *pCUC3*:*CFP* promoter activity in wild-type (WT), *mir164a-4*, *CUC2g-m4* and *ago1-27* backgrounds. The promoter activity is evaluated by quantifying fluorescence levels in developing first teeth for blade length between 400 and 600μm, sinus number *n* ≥ 8. Data are represented as boxplots.
(E) Dissection index of leaves 11, 12 and 13 between 750 and 1250 μm long of WT, *cuc3-105*, *CUC2g-m4*, *CUC2g-m4 cuc3-105*, *mir164a-4*, *mir164a-4 cuc3-105* (for each genotype, leaf number *n* ≥8).
Scale bar: 20μm.
(PDF)

S5 Fig Detailed characterization of the auxin response during CUC2-induced tooth development, related to Fig 6. (A-B) Dynamics of *DII-VENUS* (A), *mDII-VENUS* (B) after an 8h ethanol induction. Time following the start of induction is indicated. (A) 48h after induction, arrowhead shows local DII-VENUS degradation, reflecting increased early auxin signaling. Local depletion of DII-VENUS is clearly visible until 96h after induction but starts to become fainter at 127h. (B) In contrast to DII-VENUS, mDII-VENUS distribution remains uniform throughout observation period. Note that the 0h time-point corresponds to an un-induced control.
(C) Quantification of *pDR5*:*VENUS* activity in wild type (WT), *mir164a-4*, *CUC2g-m4* and *ago1-27* backgrounds. The promoter activity is evaluated by quantifying fluorescence levels in developing first teeth for blade length between 400 and 600μm, sinus number *n* ≥ 8. Data are represented as boxplots.
(D) Modification of the *pDR5*:*VENUS* pattern after 10μM NPA treatment in uninduced *CUC2i*. Time following NPA treatment is indicated. White arrowheads point to *pDR5*:*VENUS* signal outside of vasculature 3 hours after NPA application. Note that the *pDR5*:*VENUS* signal tends to be homogeneous 10h after NPA treatment.
(E) *pDR5*:*VENUS* expression following NPA application at 24h (NPA @24h) or 48h (NPA @48h) after an 8h ethanol induction in *CUC2i* observed 96h after induction start.
(F) Scoring of *pDR5*:*VENUS* maxima in a *CUC2i* background after an 8h ethanol induction and following NPA applications. Three classes, with increasing size and intensity of the *pDR5*:*VENUS* expressing domain are defined and used to score *pDR5*:*VENUS* maxima observed 96h after ethanol induction start and following NPA treatments @24h or @48h. Sample size is indicated in the bars.
(G) *pDR5*:*VENUS* expression following NPA application at 48h (NPA @48h), 72h (NPA @72h) or 96h (NPA @96h) after an 8h ethanol induction in a *CUC2i* observed 124h after induction start.
(H) Scoring of *pDR5*:*VENUS* maxima in a *CUC2i* background after an 8h ethanol induction and following NPA applications. Scoring was done as in (E) except that the observations were made 124h after the start of the ethanol induction instead of 96h.
(I,J) Blade length measured 168h after an 8h ethanol induction combined with early (I) and late (J) NPA applications. Data are mean ± SEM, for each treatment leaf number is *n* ≥ 14. Letters show treatments with no significant difference (p-value<0.01) in one-way ANOVA followed by Tukey’s HSD.
(K, L) Sinus angle measured 168h after an 8h ethanol induction combined with early (K) and late (L) NPA applications. Data are mean ± SEM, for each treatment sinus number is *n* ≥ 22. Letters show treatments with no significant difference (p-value<0.01) in one-way ANOVA followed by Tukey’s HSD.
(M,N) Tooth observed respectively 96h (L) and 124h (M) after an 8h ethanol induction combined with NPA applications.
Scale bars: (A, B, D) 20μm, (E, G, M) 50μm and (N) 100μm.
(PDF)

S6 Fig Detailed characterization of *KLUH* as a functionnal relay for CUC2-triggered toth outgrowth, related to Fig 7. (A) Relative localization of RFP-CUC2 protein and expression of a *pKLUH*:*GFP* reporter after an 8h ethanol induction. *pKLUH-GFP* is observed in the center of the RFP-CUC2 domain.
(B) Sinus angle dynamics after an 8h ethanol induction in a *CUC2i* and *CUC2i kluh-4* background. Data are mean ± SEM, sinus number *n* ≥ 41. Statistical significance (Student’s test) is designated by * p<0.05, *** p<0.005.
(C) Real-time RT-PCR quantification of *KLUH* mRNA levels in WT, *cuc2-3* and *CUC2g-m4* plants. RNAs were extracted from 2-week-olds whole seedlings grown in vitro in long-day conditions and expression levels were normalized by *EF1α* and *qREF*. Crosses represent individual data points while squares are mean of the different samples, sample number *n* = 3.
(D) Dissection index of leaves 11, 12 and 13 between 750 and 1250 μm long of WT, *kluh-4*, *CUC2g-m4*, *CUC2g-m4 kluh-4*, *mir164a-4*, *mir164a-4 kluh-4* (for each genotype leaf number *n* ≥8).
Scale bars: 20μm.
(PDF)

S7 Fig Contribution of the strong local auxin response to the dynamics of *pCUC3:CFP*, *pKLUH:GFP* and RFP-CUC2 expression, related to Fig 9. (A) RFP-CUC2 distribution and *pDR5*:*VENUS* expression pattern observed 52h after the start of a 2x8h ethanol induction followed by NPA application @0h and @24h relative to induction. For the *RFP-CUC2* and *pDR5*:*VENUS* channels, pixel intensity is represented with the Fire LUT. We used here a double induction of 2x8h to extend the duration of RFP-CUC2 expression and allow imaging at 52h. In mock treatment, a clear *pDR5*:*VENUS* maximum is visible at the leaf margin, while this maximum is absent in NPA-treated leaves. In mock-treated leaves, RFP-CUC2 is distributed into two domains (arrowheads) separated by a central domain with lower RFP-CUC2, corresponding to the zone of pDR5:VENUS maximum. In NPA-treated plants, this RFP-CUC2 distribution discontinuity is not observed.
(B) *pKLUH*:*GFP* expression observed 48h after an 8h ethanol induction in a *CUC2i* background and following NPA application 24h (NPA @24h) after ethanol induction start. Pixel intensity is represented with the Fire LUT.
Scale bars: (A) 20μm, (B) 50μm.
(PDF)

S1 Table Acquisition parameters for confocal reporter imaging. (PDF)

S2 Table Primers used for real-time PCR analysis. (PDF)

S1 File Spreadsheet with the numerical data underlying graphs from the main figures. (XLSX)

S2 File Spreadsheet with the numerical data underlying graphs from the supporting information figures. (XLSX)
