# Supplementary figures and images for: Dissecting the pathways coordinating patterning and growth by plant boundary domains
Source: PLoS Genet. 2019 Jan 24;15(1):e1007913. doi: 10.1371/journal.pgen.1007913 (PMC6363235; doi:10.1371/journal.pgen.1007913)

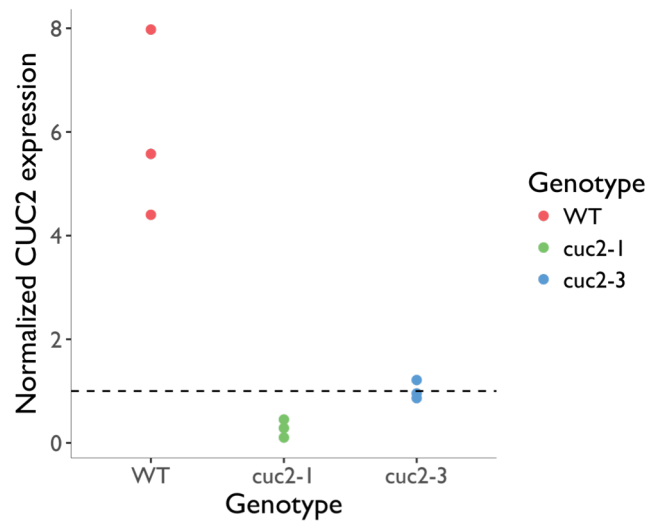

Supplement: S1 Fig — Real-time RT-PCR quantifications of CUC2 mRNA levels in the wild type (WT), cuc2-1 and cuc2-3 mutants. Total RNAs were extracted from 2 week-old plants dissected to remove all leaves and CUC2 mRNA levels were normalized by EF1α and qREF. Each point represents a biological replicate. (PDF) [file pgen.1007913.s001.pdf]

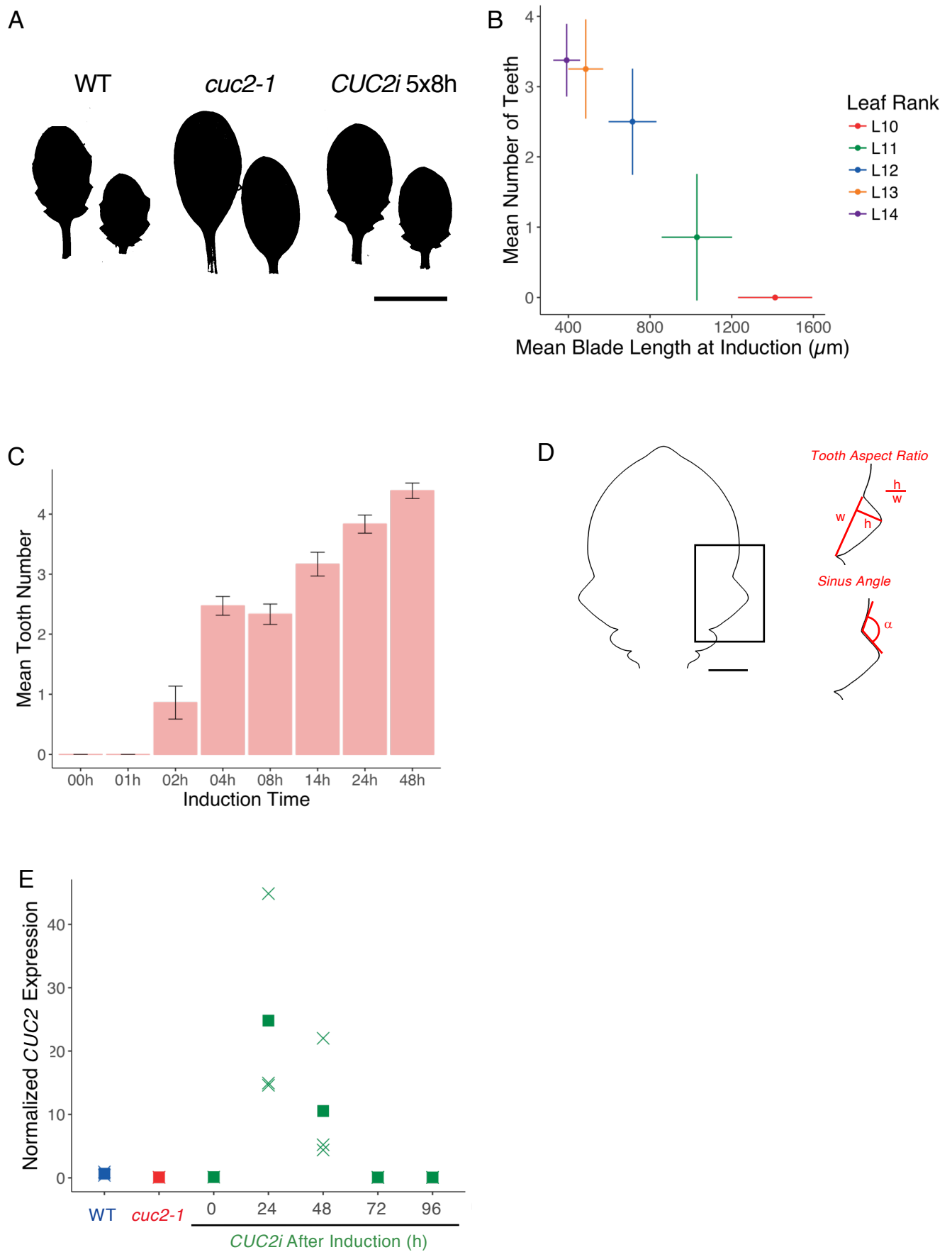

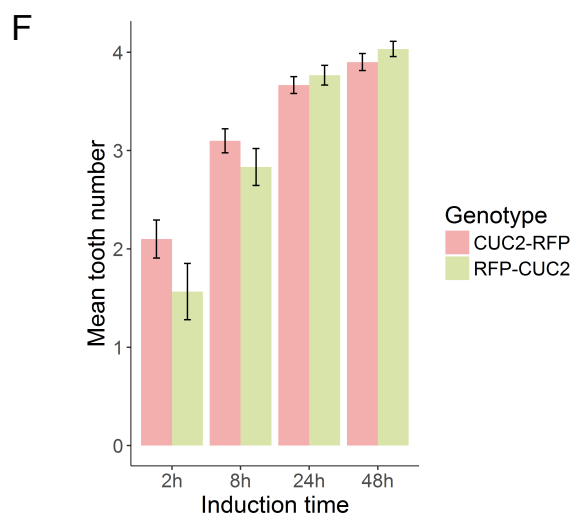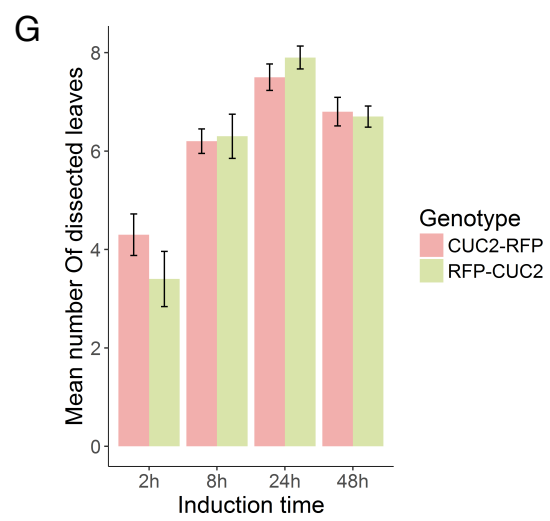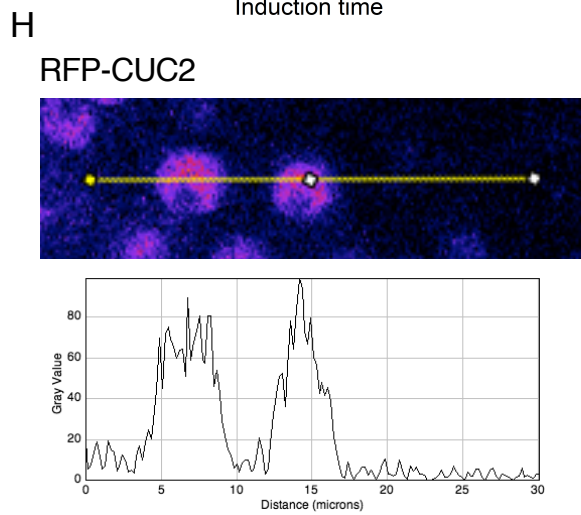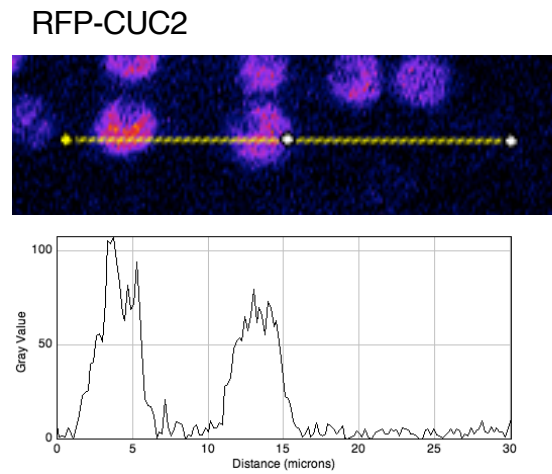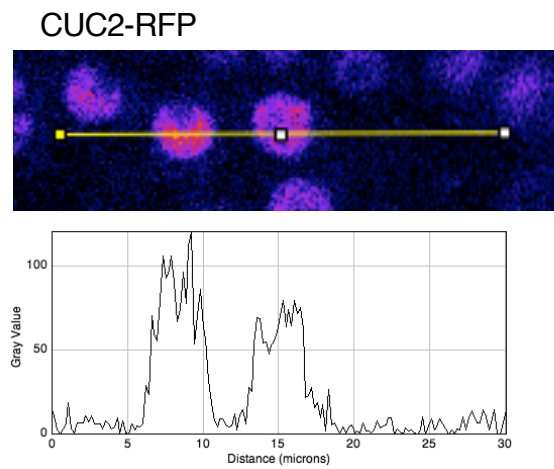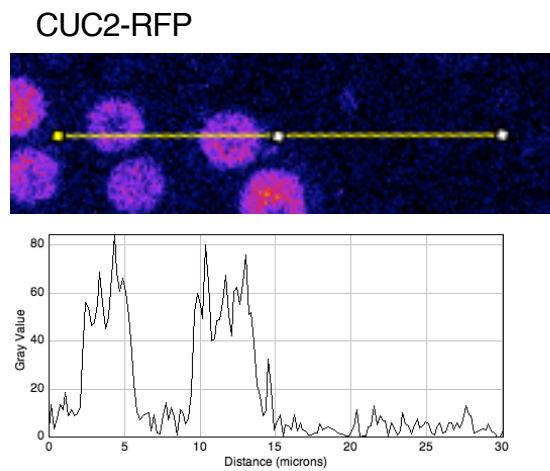

Supplement: S2 Fig — (A) Prolonged CUC2i induction restores multiple teeth formation. Leaf silhouettes from leaves of comparable rank from wild type (WT), cuc2-1 and CUC2i plants induced by ethanol for 5x8h and observed 9 days after induction. (B) Only small leaf primordia (< ∼1200μm) form teeth following CUC2i induction. The primordia size of leaves L10 to L14 was measured at the induction start in half of the plants. The number of teeth formed following a 6h ethanol induction on L10 to L14 was determined one week after induction on the other half of the plants. Data are mean ± SD, leaf number n ≥ 8. (C) CUC2i induction duration determines the number of teeth formed. Data represent mean ± SEM (leaf number n = 12) of teeth number formed following 1h to 48h ethanol inductions. Teeth were counted one week after the induction start on the three most dissected leaves. (D) Representation of the Tooth Aspect Ratio that is defined as the tooth height (h) / tooth width (w) ratio. It quantifies anisotropic growth and integrates both growth promotion at the tip and growth repression at the sinus. Representation of the Sinus Angle (α) measured in the distal sinus of the first tooth. It is a local parameter more directly related to the local growth repression in the sinus. (E) CUC2 mRNA is detected for two days following an 8h ethanol induction. Real-time RT-PCR quantifications of CUC2 expression in the WT, cuc2-1 and CUC2i at 0 to 96 hours after an 8h ethanol induction. RNAs were extracted from microdissected leaf margins and CUC2 levels are normalized by EF1α and qREF. Crosses represent individual data points while squares are mean of the different samples, sample number n ≥ 2. (F-G) The CUC2i line expressing a RFP-CUC2 fusion shows a response to varying durations of ethanol induction similar to that of a line expressing a CUC2:RFP fusion. (F) Data are mean ± SEM (leaf number n = 10) of teeth number formed following 2h to 48h ethanol inductions and (G) the mean ± SEM of the number of leaves showi [file pgen.1007913.s002.pdf]

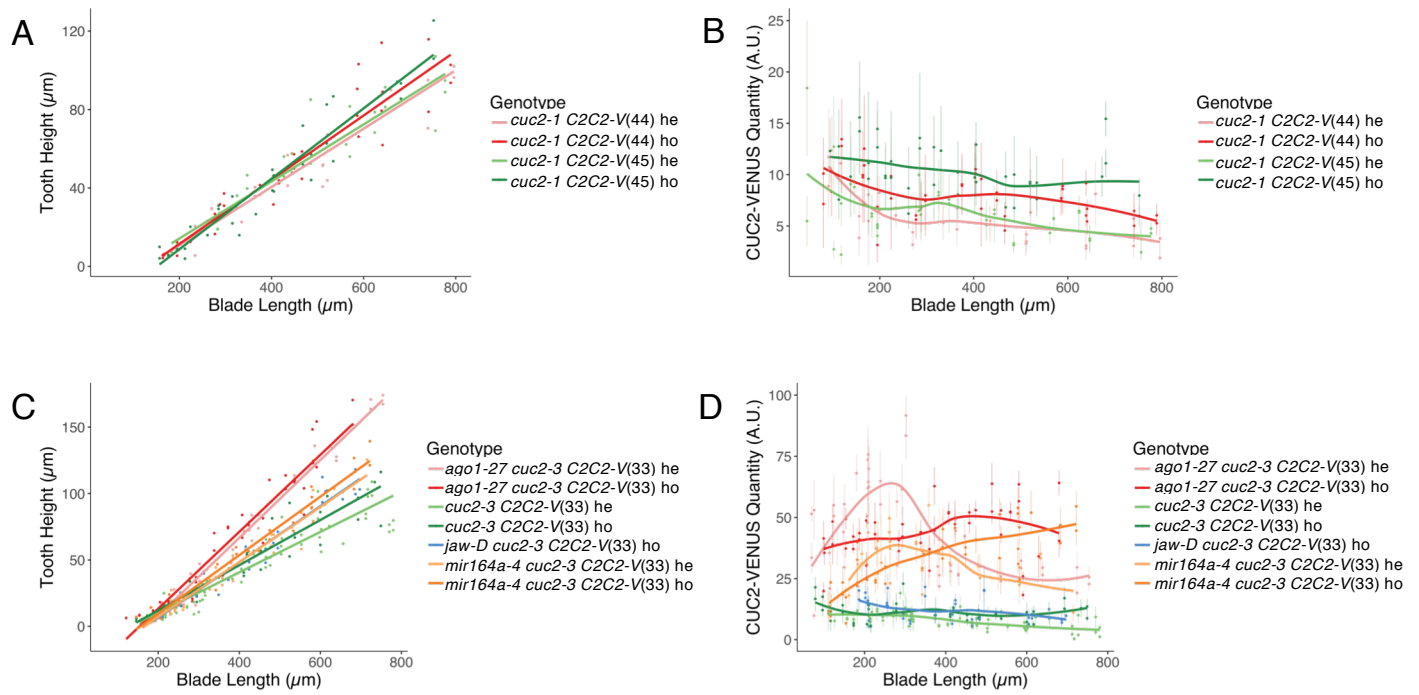

Supplement: S3 Fig — (A,C) Tooth height evolution along blade length in different genotypes. Data are individual measures and a linear regression for each genotype is shown (for all genotypes r > 0.93). The regression slope is the Tooth growth rate in Fig 3. (B,D) Quantification of CUC2-VENUS fluorescence and local regression during leaf development, each point is the mean ± SD of n = 12 nuclei per sinus. The grey area limits the interval used to calculate mean CUC2 quantity in Fig 3. (PDF) [file pgen.1007913.s003.pdf]

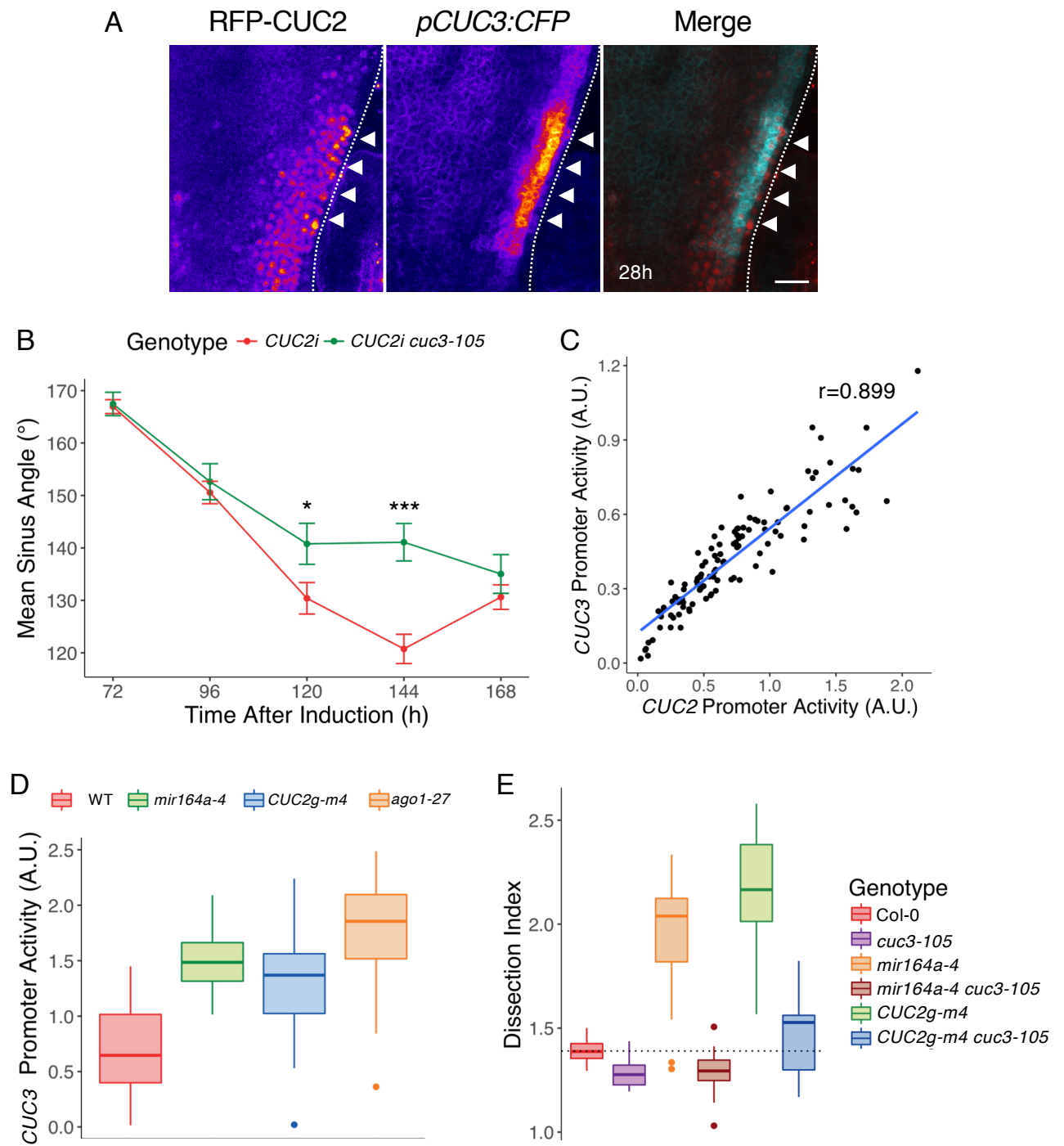

Supplement: S4 Fig — (A) Relative localization of RFP-CUC2 protein and expression of a pCUC3:CFP reporter after an 8h ethanol induction. The time following the start of induction is shown on the overlay panels. Note coexpression of RFP-CUC2 and pCUC3:CFP in the epidermis (arrowheads in A). (B) Sinus angle dynamics after an 8h ethanol induction in a CUC2i and CUC2i cuc3-105 background. Data are mean ± SEM (sinus number n ≥ 10). Statistical significance (Student’s test) is designated by * p<0.05, *** p<0.005. (C) Correlation between pCUC2:RFP and pCUC3:CFP promoter activity in a wild-type background. The promoter activity is evaluated by quantifying fluorescence levels in developing first teeth for blade length <1000 μm. Data are represented as individual measures and a linear regression (r = 0.899). (D) Quantification of pCUC3:CFP promoter activity in wild-type (WT), mir164a-4, CUC2g-m4 and ago1-27 backgrounds. The promoter activity is evaluated by quantifying fluorescence levels in developing first teeth for blade length between 400 and 600μm, sinus number n ≥ 8. Data are represented as boxplots. (E) Dissection index of leaves 11, 12 and 13 between 750 and 1250 μm long of WT, cuc3-105, CUC2g-m4, CUC2g-m4 cuc3-105, mir164a-4, mir164a-4 cuc3-105 (for each genotype, leaf number n ≥8). Scale bar: 20μm. (PDF) [file pgen.1007913.s004.pdf]

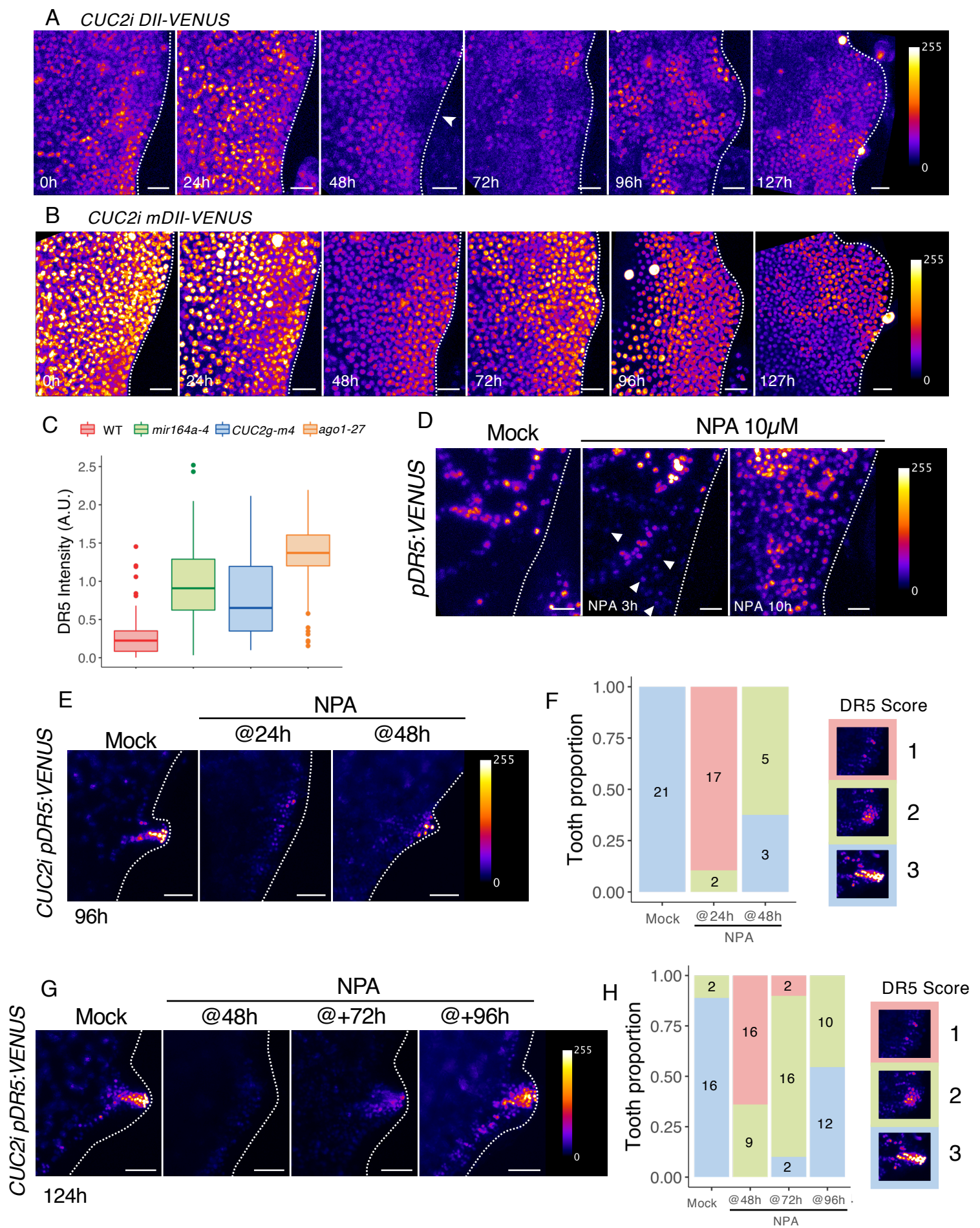

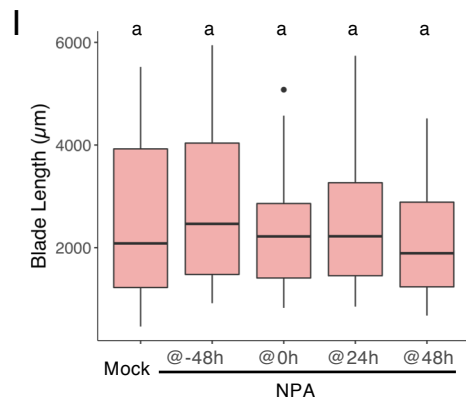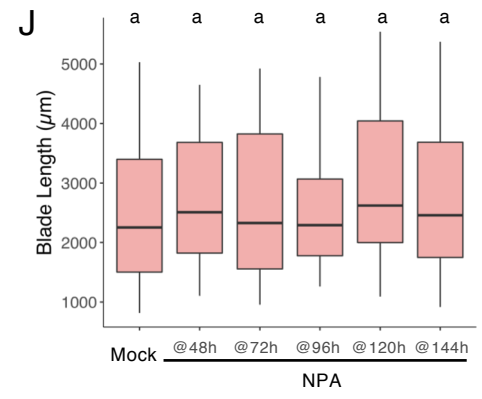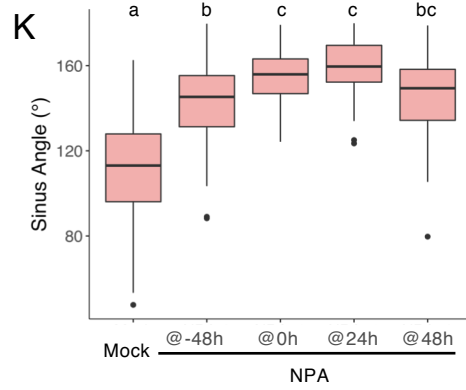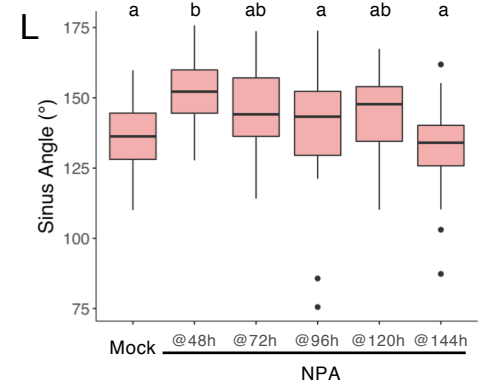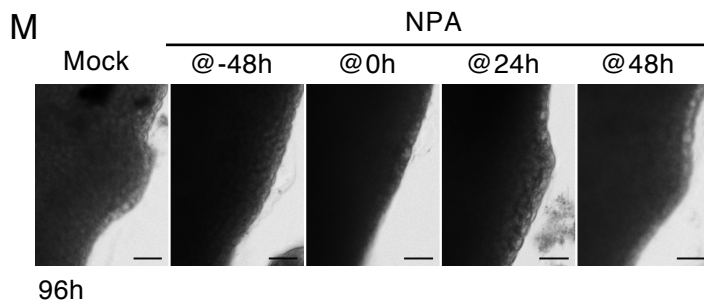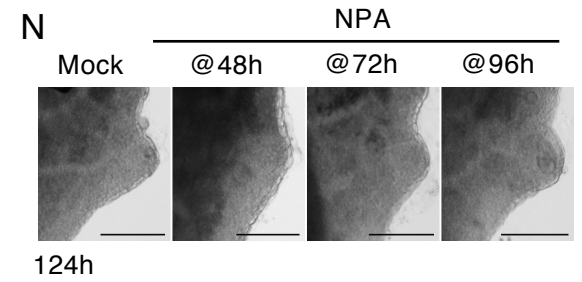

Supplement: S5 Fig — (A-B) Dynamics of DII-VENUS (A), mDII-VENUS (B) after an 8h ethanol induction. Time following the start of induction is indicated. (A) 48h after induction, arrowhead shows local DII-VENUS degradation, reflecting increased early auxin signaling. Local depletion of DII-VENUS is clearly visible until 96h after induction but starts to become fainter at 127h. (B) In contrast to DII-VENUS, mDII-VENUS distribution remains uniform throughout observation period. Note that the 0h time-point corresponds to an un-induced control. (C) Quantification of pDR5:VENUS activity in wild type (WT), mir164a-4, CUC2g-m4 and ago1-27 backgrounds. The promoter activity is evaluated by quantifying fluorescence levels in developing first teeth for blade length between 400 and 600μm, sinus number n ≥ 8. Data are represented as boxplots. (D) Modification of the pDR5:VENUS pattern after 10μM NPA treatment in uninduced CUC2i. Time following NPA treatment is indicated. White arrowheads point to pDR5:VENUS signal outside of vasculature 3 hours after NPA application. Note that the pDR5:VENUS signal tends to be homogeneous 10h after NPA treatment. (E) pDR5:VENUS expression following NPA application at 24h (NPA @24h) or 48h (NPA @48h) after an 8h ethanol induction in CUC2i observed 96h after induction start. (F) Scoring of pDR5:VENUS maxima in a CUC2i background after an 8h ethanol induction and following NPA applications. Three classes, with increasing size and intensity of the pDR5:VENUS expressing domain are defined and used to score pDR5:VENUS maxima observed 96h after ethanol induction start and following NPA treatments @24h or @48h. Sample size is indicated in the bars. (G) pDR5:VENUS expression following NPA application at 48h (NPA @48h), 72h (NPA @72h) or 96h (NPA @96h) after an 8h ethanol induction in a CUC2i observed 124h after induction start. (H) Scoring of pDR5:VENUS maxima in a CUC2i background after an 8h ethanol induction and following NPA applications. Scoring was done as in (E) except [file pgen.1007913.s005.pdf]

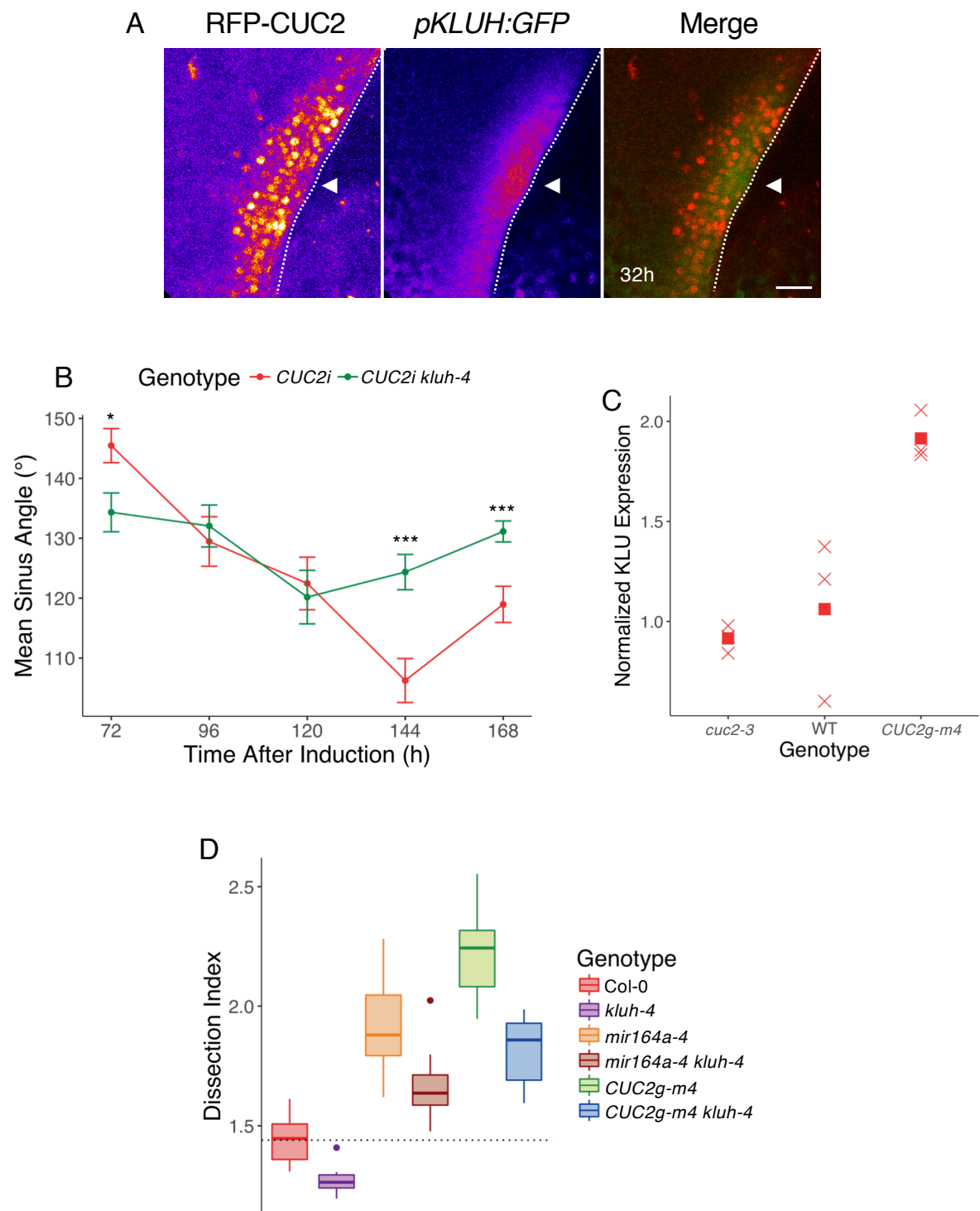

Supplement: S6 Fig — (A) Relative localization of RFP-CUC2 protein and expression of a pKLUH:GFP reporter after an 8h ethanol induction. pKLUH-GFP is observed in the center of the RFP-CUC2 domain. (B) Sinus angle dynamics after an 8h ethanol induction in a CUC2i and CUC2i kluh-4 background. Data are mean ± SEM, sinus number n ≥ 41. Statistical significance (Student’s test) is designated by * p<0.05, *** p<0.005. (C) Real-time RT-PCR quantification of KLUH mRNA levels in WT, cuc2-3 and CUC2g-m4 plants. RNAs were extracted from 2-week-olds whole seedlings grown in vitro in long-day conditions and expression levels were normalized by EF1α and qREF. Crosses represent individual data points while squares are mean of the different samples, sample number n = 3. (D) Dissection index of leaves 11, 12 and 13 between 750 and 1250 μm long of WT, kluh-4, CUC2g-m4, CUC2g-m4 kluh-4, mir164a-4, mir164a-4 kluh-4 (for each genotype leaf number n ≥8). Scale bars: 20μm. (PDF) [file pgen.1007913.s006.pdf]

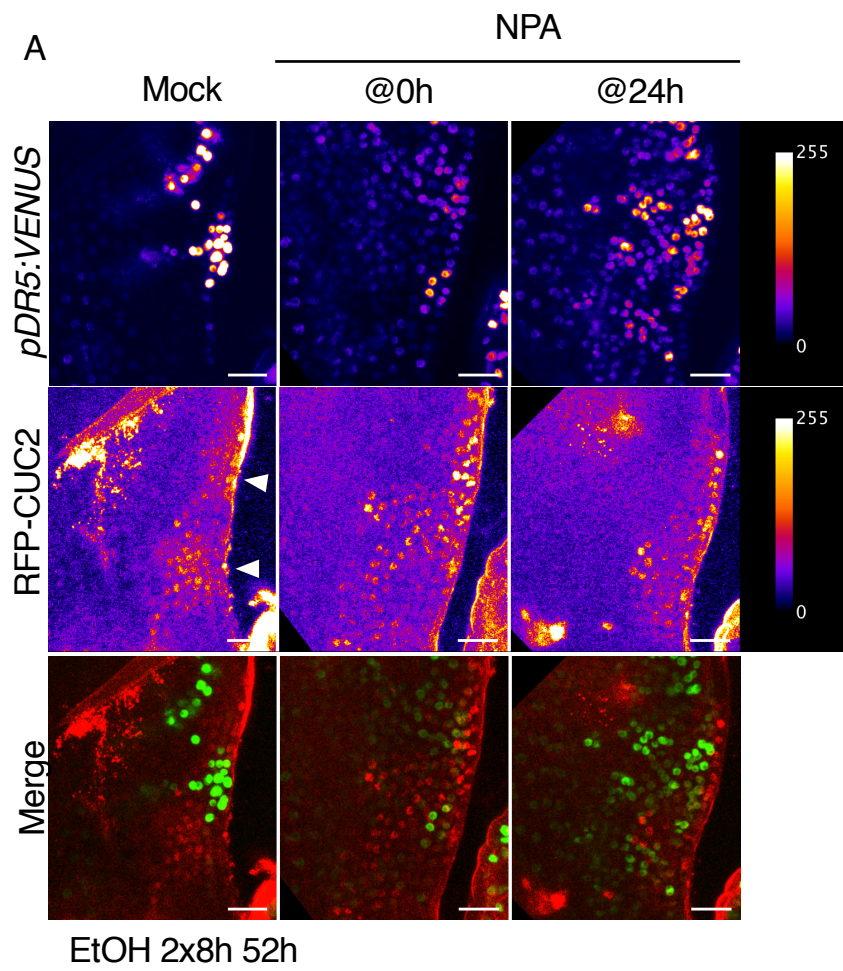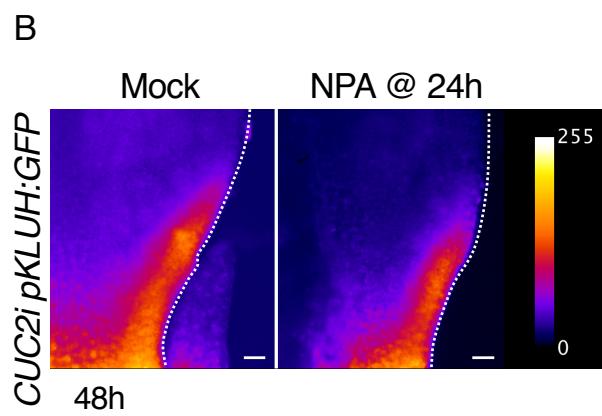

Supplement: S7 Fig — (A) RFP-CUC2 distribution and pDR5:VENUS expression pattern observed 52h after the start of a 2x8h ethanol induction followed by NPA application @0h and @24h relative to induction. For the RFP-CUC2 and pDR5:VENUS channels, pixel intensity is represented with the Fire LUT. We used here a double induction of 2x8h to extend the duration of RFP-CUC2 expression and allow imaging at 52h. In mock treatment, a clear pDR5:VENUS maximum is visible at the leaf margin, while this maximum is absent in NPA-treated leaves. In mock-treated leaves, RFP-CUC2 is distributed into two domains (arrowheads) separated by a central domain with lower RFP-CUC2, corresponding to the zone of pDR5:VENUS maximum. In NPA-treated plants, this RFP-CUC2 distribution discontinuity is not observed. (B) pKLUH:GFP expression observed 48h after an 8h ethanol induction in a CUC2i background and following NPA application 24h (NPA @24h) after ethanol induction start. Pixel intensity is represented with the Fire LUT. Scale bars: (A) 20μm, (B) 50μm. (PDF) [file pgen.1007913.s007.pdf]
