# Supplementary material for: Dissecting the pathways coordinating patterning and growth by plant boundary domains
Source: PLoS Genet. 2019 Jan 24;15(1):e1007913. doi: 10.1371/journal.pgen.1007913 (PMC6363235; doi:10.1371/journal.pgen.1007913)
Supplement: S1 Table — (PDF) [file pgen.1007913.s008.pdf]

Reporter	Detector type	Excitation wavelength (nm)	Detection wavelength (nm)
pCUC2:CUC2-VENUS	Hybrid	514	530-580
RFP-CUC2	Hybrid	561	590-631
pCUC3:CFP	Hybrid	458	470-512
pKLUH:GFP, pPIN1:PIN1-GFP	Hybrid	488	498-535
DII-VENUS	Hybrid	514	520-555
mDII-VENUS, pDR5:VENUS	PMT	514	520-555
