# Supplementary material for: Dissecting the pathways coordinating patterning and growth by plant boundary domains
Source: PLoS Genet. 2019 Jan 24;15(1):e1007913. doi: 10.1371/journal.pgen.1007913 (PMC6363235; doi:10.1371/journal.pgen.1007913)
Supplement: S2 Table — (PDF) [file pgen.1007913.s009.pdf]

Primer Name	Sequence (5'-3')	Reference
qEF1a-F	CCTTGGTGTCAAGCAGATGATC	
qEF1a-R	GATTTTGTCGGGGTTGTAACCAAC	
qREF-F	AACTCTATGCAGCATTTGATCCACT	(Morineau, et al., 2016)
qREF-R	TGATTGCATATCTTTATCGCCATC	(Morineau, et al., 2016)
qCUC2-F	CTTGGCAACTTCCCGGGAGA	(Tian, et al., 2014)
qCUC2-R	CCAGCCTCAGTTGCTCTGTTAGTT	(Tian, et al., 2014)
qCUC3-F	GGCGGAGGAGGACAGCTTGTT	
qCUC3-R	TGAGGCCACGTGGAGCCCTA	
qKLUH-F	GGTGTAGAGCTTTGGTCTCTGAAG	
qKLUH-R	GATTATTACCCTTCTTCATCTTGTG	

Morineau C, Gissot L, Bellec Y, Hematy K, Tellier F, Renne C, et al. Dual Fatty Acid Elongase Complex Interactions in Arabidopsis. PLoS ONE. 2016;11(9):e0160631. doi: 10.1371/journal.pone.0160631.

Tian C, Zhang X, He J, Yu H, Wang Y, Shi B, et al. An organ boundary-enriched gene regulatory network uncovers regulatory hierarchies underlying axillary meristem initiation. Mol Syst Biol. 2014;10:755. doi: 10.15252/msb.20145470.
